# Statistical analysis for heat transfer optimization of magnetohydrodynamics trihybrid nanofluid over a convectively heated Riga surface

**DOI:** 10.1038/s41598-025-32787-0

**Published:** 2025-12-19

**Authors:** Maher Jebali, Sohail Rehman, Mohamed Bouzidi, Muhammad Eisa, Samia NASR, Bilal Himmat

**Affiliations:** 1 Computer Science Department, Applied College, University of Hail, P.O. Box 2440, 55476 Ha’il City, Saudi Arabia; 2 Department of Physical and Numerical Sciences, Qurtuba University of Science and Information Technology, Peshawar, 25000 KP Pakistan; 3 Department of Physics, College of Science, University of Ha’il, P.O. Box 2440, Ha’il, Saudi Arabia; 4 Department of Statistics, University of Peshawar, Peshawar, 25000 KP Pakistan; 5 Chemistry Department, College of Science, King Khalid University, 61413 Abha, Saudi Arabia; 6 Department of Computer Science, Faculty of Computer Science, Kabul University(KU), Kabul, Afghanistan

**Keywords:** Riga plate, Tri-hybrid nanofluid, Cross thermo-diffusion effects, Activation energy, Sensitivity analysis, Engineering, Mathematics and computing, Nanoscience and technology, Physics

## Abstract

The Riga plate is arrangement of electrodes and permanent magnets allows for efficient regulation of fluid flow. The Riga surface leverages Lorentz forces to control boundary layers (BL) and improve cooling purposes for effective electromagnetic flow control in nuclear and aeronautical engineering systems. Furthermore, by utilizing synergistic interactions of different nanoparticles, heat transfer rat can be optimized in industrial setup. The primary focus of this work is to investigate the unsteady BL flow of water-based tri-hybrid nanolfuid (tri-HNF) flow over a Riga plate senor under the influence of activation energy, cross-diffusion, and convective heating. Three different nanoparticles $A{l}_{2}{O}_{3}$, $CuO$ and $Ti{O}_{2}$ are dispersed in a pure water. The model equations are constructed using BL theory and transformed into ordinary differential equations using an appropriate similarity rule. The Runge–Kutta fourth-order (RK-4) method, along with shooting approach, is used to address the problem numerically. The skin friction and Nusselt and Sherwood numbers are assessed using optimized statistical Response Surface Methodology (RSM) technique. The Gharesim model viscosity and Hamilton-Crosser thermal conductivity models are deployed in the governing model. A mathematical model is designed and developed using RSM to obtain an optimal skin friction, heat and mass transfer rate. Sensitivity analysis (SA) is performed to investigate the response of input on these coefficients. SA shows that in narrow BL, the skin friction rises with nanoparticle concentration. Velocity of tri-HNF boost with the Hartman number and the electrode-magnet distance parameter. The Soret number, and activation energy increases the concentration profile. Higher Nusselt number indicates improved heat transfer with increased nanoparticle load. Activation energy uplift the mass transfer rates, but dwindle with nanoparticle concentration.

## Introduction

A Riga plate is an electromagnetic actuator made up of permanent magnets and alternating electrodes assembled on a horizontal surface^1^. A Riga plate concept was invented half a century ago by Gailitis and Lielausis at the Physics Institute in Riga, Latvia^2^. An arrangement of alternating electrodes and permanent magnets arranged spanwise on a flat surface constituted as a flow control device, produced wall-parallel Lorentz force^3^. This electromagnetic actuator may effectively minimize the friction and hydraulic drag in submarines through boundary layer (BL) separation mitigation and turbulence suppression. The Riga plate have potential uses including the aviation industry to reduce drag on airplane wings, in naval engineering to reduce hull friction in ships and submarines, in automotive systems to controlee the BL in high-speed automobiles, in MHD power generation for regulating flow in plasma channels, and turbomachinery for improving compressors and turbine productivity^4–7^. Due to its various applications in modern engineering it has been a core subject among modern researchers. In a recent study, the thermal hydrodynamic performance of Maxwell fluid with precise heat and mass transfer over a Riga plate was elaborated by Alrihieli et al.^8^. Their results demonstrated that when the Hartmann number is increased the Maxwell fluid displays an extremely high velocity distribution. Their study highlighted the main physical findings of improved electromagnetic influence and a reduction in the flow obstruction of the system. Sharma and Gorai^9^ examined the flow and heat transfer over a Riga squeezing plate taking melting heat at the surface. They proposed that the repercussions permit the anti-relation between melting heat and changed Hartman number in fluid flow. Magnetic behavior drives the thermal BL thickness of the Riga plate parameter expand. The dissipative nanofluid (NF) flow over a stretching Riga plate was elaborated by Ahmed et al.^10^. They further analyzed the heat and mass transfer features and shape effects. Shah^11^ investigated the bioconvection micropolar NF flow near stagnation point over tilted Riga plate implanting Keller-box approach. He suggested that velocity is increasing function of Hartman number.

The scientific community are currently interested in NF because of its importance in many applications. NF primarily involves dispersion or mixing nanoparticles with fluids, is utilized in a wide range of real-world heat transfer applications, such as cooling systems, electronic devices, biomaterials, transportation, and the food industries^12,13^. Since NF differ from their solid counterparts in terms of their physical and chemical properties, their uses in biomedical technologies has grown recently. Because of these characteristic, NF can be used for a number of biological activities, such as the splitting of magnetic cells, improving the contrast of magnetic resonance imaging, administering medication, curing infections, and causing hyperthermia, among others^14^. Tri-hybrid NF (Tri-HNF) have attracted a lot of interest because of their improved thermophysical characteristics, which result from the synergistic effects of integrating three different kinds of nanomaterials in a base fluid. These NFs are especially appealing for heat exchange applications in sectors like electronics cooling, energy generation and aerospace because they have better heat transmission capabilities, improved thermal conductivity, and enhanced stability when compared to single-phase fluids or hybrid NFs. Three different nanoparticles $A{l}_{2}{O}_{3}$, $CuO$ and $Ti{O}_{2}$ are dispersed in a water base fluid to create the tri-HNF used in this investigation. These particular nanoparticles are carefully chosen with the goal of utilizing their respective thermophysical characteristics to produce synergistic performance^15^. The nanomaterial $CuO$ offers much better thermal conductivity to dramatically increase the heat transfer capability, $Ti{O}_{2}$ helps to the stability and distribution of the mixture, and $A{l}_{2}{O}_{3}$ offers a balance between superior thermal conductivity and affordability^16,17^. An equal volumetric percentage of 5% is taken into account for each type of nanoparticle in the investigation. This method is frequently used in early research to methodically examine the combined effects of the triple hybrid composition. The enhanced performance over mono or HNF is predicted using existing models, including the Hamilton-Crosser model for thermal conductivity and the Gharesim model for viscosity.

The effect of Tri-HNF on the temporal thermal performance of inclined merge fins was investigated by Pavan et al.^18^, with an emphasis on internal heat production. Babu et al.^19^ used a statistical technique to simulate the squeezed flow of HNF made of PEG and water over a magnetic sensor surface, providing information on the scattering of nanoparticles in intelligent sensing systems. Rauf et al.^20^ investigated convective heat transfer in a non-Newtonian Tri-HNF with non-local kernel conditions and rising temperatures. The Tri-HNF technique performs better than hybrid and traditional NF in terms of energy transfer and momentum profile, according to their numerical and graphical evaluations. On flat and thin sheets, Olabode et al.^21^ thoroughly examined Tri-HNF flow with temperature-dependent thermophysical characteristics. Using Keller box analysis, Shah^11^ investigated the stagnation-point flow of micropolar bioconvective nanofluids over a tilted Riga plate, highlighting the significance of magnetohydrodynamic (MHD) phenomena in microscopic heat transfer. A growing curiosity in simulating and optimizing tri-HNF systems under diverse physical conditions is highlighted by these research taken together.

Density variations brought on by the temperatures, chemical, and concertation gradients driven flow occurs in many natural transportation mechanisms^22,23^. Therefore, it is significant to investigate flow caused by concentration variations separately or in conjunction with temperature variations. The Dufour effect is a physical phenomenon arises due to heat flux brought on by the chemical composition gradient (diffusion-thermo). However, if temperature gradients are the source of mass fluxes, this phenomenon is known as the thermal-diffusion (Soret effect)^24^. In most cases, these effects are only an insignificant order of magnitude. When these effects are taken jointly they are referred to as cross-diffusion effects. The cross-diffusion effects in a three dimensional mixed convection flow of Maxwell fluid embedded in a Darcy-Forchheimer medium were elaborated by Zhnag et al.^25^. They verified that when diffusion thermal effects and thermos-diffusion are increased, the temperature and concentration behave in opposing ways. Estimation of cross diffusion phenomenon on the chemically reacting micropolar fluid flow over an extending sheet was explored by Salawu^26^. It is clearly demonstrated that the concentration profile decreases with chemical reaction, while the cross diffusion influences enhance the system heat dispersion. An unsteady bioconvective fluid flow across concentric elongating cylinders with cross-diffusion effects were scrutinized by Shaheen et al.^27^. They concluded the conclusion that the thermal field expands by increasing the thermal conjugate parameter and Dufour number. When the Soret and Dufour numbers are amplified, the heat and mass flux shows reverse behavior. The computational investigation of cross-diffusion effects of chemically reactive magneto-NF over an oscillating absorbent plate were explored by Reddy et al.^28^. As cross diffusion and radiation effects increase, the temperature distribution widens. The concentration distribution is expanded by the thermo-diffusion effect. Zhao et al.^29^ examined the free-convective flow of NF due to titled surface taking cross-diffusion effects and chemical reaction. The cross-diffusion and thermal radiation effects on MHD non-Newtonian BLF over two different morphologies were investigated by Dharmaiah et al.^30^. They discovered that the temperature field improves with an increase in the diffusion thermal parameter. Temperature and velocity increase and concentration falls as radiation absorption increases. The radiation intake parameter decreases the concentration and increases temperatures closer to the porous boundary layer (BL).

Sensitivity analysis (SA) together with Response Surface Methodology (RSM) is commonly employed in aerodynamics for the optimization of drag and lift coefficients and in fluid dynamics to enhance parameters such as Reynolds number, concentration of nanoparticles, and strength of magnetic field in NF heat transfer systems. Beyond fluid mechanics, RSM-based SA propels advances in medical device engineering such as delivery of medications, materials science composite to optimize material design, and energy systems for fuel cell performance tuning, allowing for data-driven decision-making at low computational cost. In this regard, an active parametric SA to develop electrical and thermal exergy/energy proficiency of PVT system using NF was elaborated by Jabeen et al.^31^. They found that the goal of simultaneously achieving optimal electrical and thermal power is positively impacted by multiple optimization objectives with the ideal composite acceptability values, which illustrates the impact of input design parameters. Lin et al.^32^ performed SA for an investigation of hybrid thermal/photovoltaic system based on intense NF. They concluded that the solar radiation is among the most sensitive factor for electrical performance, with R and F-values of 10.729 and 2934.770, respectively. The R-value and F-value for solar radiation are 27.620 and 13,689.811, respectively, for energy efficiency, demonstrating its substantial impact. The heat transfer optimization using SA in buoyancy driven flow of Williamson tri-HNF over a thin needle was conducted by Bouzidi et al.^33^. The found that the higher load 5% of nanomaterial is more sensitive to heat transfer. Huang et al.^34^ performed SA for thermal conductivity of Aluminum-water based NF. According to their findings, the utilization of sphere-shaped particles enables the NF thermal conductivity to be stable over time, and the sensitive of volume fraction section can result in thermal conductivity fluctuating across a wide range ranges from 2.5% and 5.5%. A novel neural network and SA for forecasting the thermal confrontation of heat pipes with NF was proposed by Wang et al.^35^. They arrived at the conclusion that this work offers clear guidelines for creating a highly accurate and universal forecasting model for heat pipes using NF.

Numerous researchers have used single-objective heat transfer enhancement under the impacts of MHD and heat radiation in a BLF over a Riga sensor. However, these models usually fails inadequately in capturing interaction effects such as cross-diffusion effects, activation energy, and bi-convection effects in a BLF. The BLF of tri-HNF and heat transfer optimization using regression and sensitivity analysis, which is significant in engineering technologies and thermal management using tri-HNF, is examined for the first time. A tri-HNF containing three distinct different nanoparticles $A{l}_{2}{O}_{3}$, $CuO$ and $Ti{O}_{2}$ are used in the study to create an improved cooling fluid with improved heat transfer capabilities. The Riga plate sensor is a popular technique used in marine and aeronautical engineering to manipulate the BLF in order to increase efficiency and decrease drag. The use of statistical and computational methods (SA and RSM) are used to develop a framework to forecast and optimize the system performance. The current study combines the RSM with a computational method RK-4 to formulate and prediction framework that assesses skin friction, heat transfer, and mass transfer performance under various physical parameters simultaneously. A state-of-the-art development in thermal fluid technology, this numerical solution for the Riga plate-induced momentum, thermal, and concentric BL, this study grasp a critical research gap. These solutions are informative for future Riga plate research, not only for verification purposes but also for detecting novel physical properties because of their simplicity and elegance in the wall shear, heat, and mass transfer expressions.

Problem statement and analysis.

**Fig. 1:**
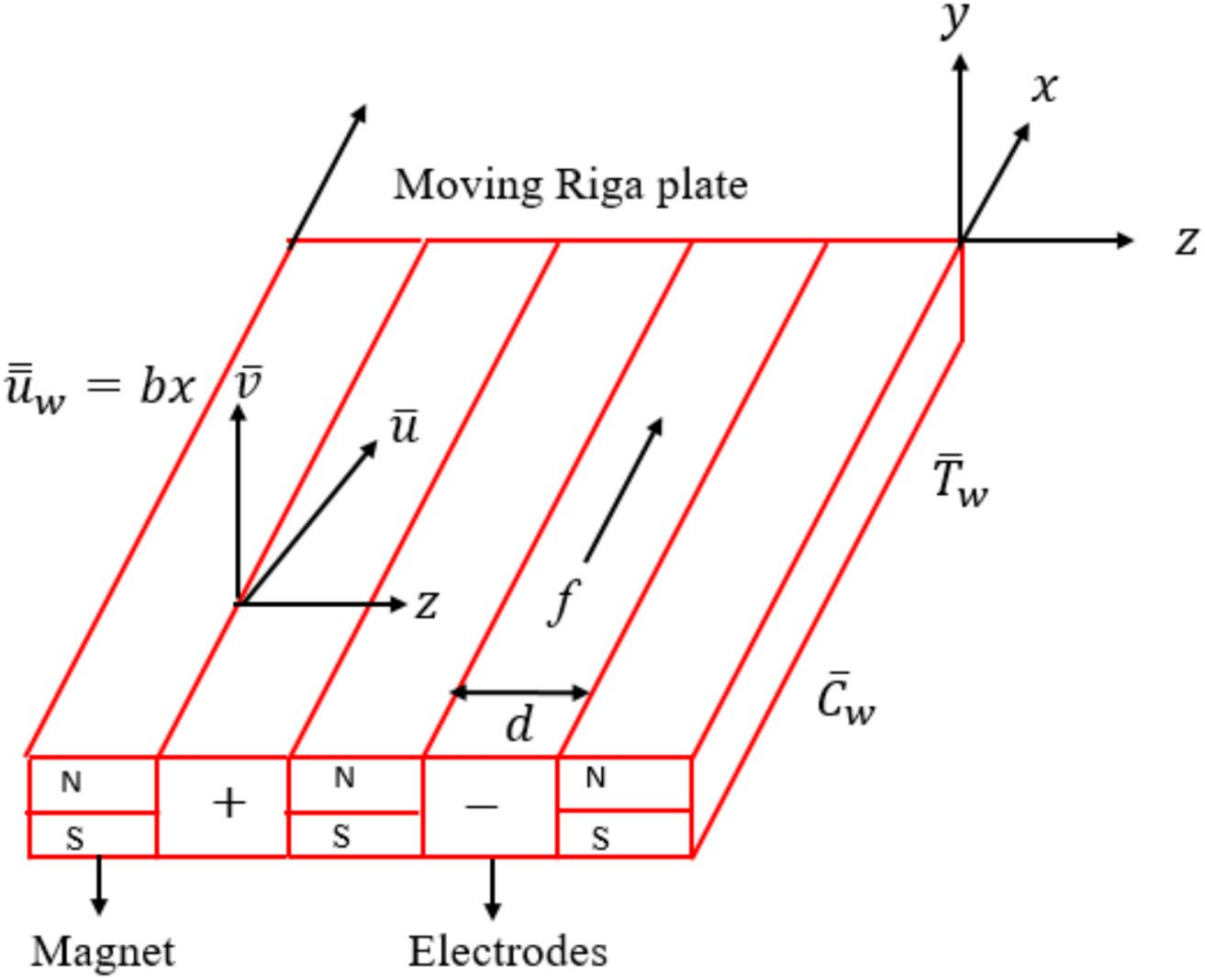
Illustration of proposed model and coordinates system.

The aim of this analysis is to model and investigate the electro-magneto-hydrodynamic mechanisms in an unsteady BLF of water-based tri-HNF across a Riga plate. A laminar time-dependent fluid flow is generated across a convectively heated Riga plate is described in a Cartesian coordinate system $\left(x,y\right).$ An infinite Riga plate stretches in $x-$ direction and $y-$ axis is normal to it, with $u$ and $v$ denoting velocity components in $x$ and $y-$ directions, respectively. The Riga plate electromagnetic field induces an external parallel Lorentz force. As the distance normal to the plate increases, this force decreases significantly. As illustrated in Fig. 1, the Riga-plate is constructed from a permanent magnet and an alternating arrangement of electrodes positioned on a horizontal surface separated by a distance $d$. Only a Lorentz force $f=J\times B$, aligned to the arrangement accelerate the flow over the horizontal plate with ${\overline{\overline{u}}}_{w}=bx$, where $b>0$ is constant. There are no additional driving forces (such as an expanding wall or pressure gradient) besides to this electromagnetic body force. The velocity ${\overline{\overline{u}}}_{e}\to cx$ describes the fluid that is far from the plate. The plate face comes into contact with water-based tri-HNF, at temperature ${\overline{T} }_{w}$ and concentration ${\overline{C} }_{w}$, at the surface with heat and mass transfer coefficient ${H}_{f}$ and ${H}_{m}$, respectively. Assuming zero typical flux through the plate dynamically regulates the concentration of nanoparticles there. The cross-diffusion effects, activation energy, and bi-convection effects are assumed in the model.

The governing BL equations can be expressed in the following ways by using the Oberbeck-Boussinesq approximation^5,36–38^:

1 $$\frac{{\partial \overline{u}}}{\partial x} + \frac{{\partial \overline{v}}}{\partial y} = 0$$

2 $$\left( {\frac{{\partial \overline{u}}}{\partial t} + \overline{u}\frac{{\partial \overline{u}}}{\partial x} + \overline{v}\frac{{\partial \overline{u}}}{\partial y}} \right) = \frac{{\partial \overline{\overline{u}}_{e} }}{\partial t} + \frac{{\partial \overline{\overline{u}}_{e} }}{\partial x} + \frac{{\mu_{tri - HNF} }}{{\rho_{tri - HNF} }}\frac{{\partial^{2} \overline{u}}}{{\partial y^{2} }} + \frac{{\pi J_{0} M_{0} }}{{8\rho_{tri - HNF} }}e^{{ - \left( {\frac{\pi }{d}} \right)y}}$$

3 $$\left( {\frac{{\partial \overline{T}}}{\partial t} + \overline{u}\frac{{\partial \overline{T}}}{\partial x} + \overline{v}\frac{{\partial \overline{T}}}{\partial y}} \right) = \frac{{k_{tri - HNF} }}{{\left( {\rho c_{p} } \right)_{tri - HNF} }}\frac{{\partial^{2} \overline{T}}}{{\partial y^{2} }} + \frac{{16\sigma^{*} }}{{3\left( {\rho c_{p} } \right)_{tri - HNF} k^{*} }}\frac{{\partial^{2} \overline{T}}}{{\partial y^{2} }} + \frac{{D_{m} K_{T} }}{{\left( {c_{p} } \right)_{tri - HNF} C_{S} }}\frac{{\partial^{2} \overline{C}}}{{\partial y^{2} }}$$

4 $$\left( {\frac{{\partial \overline{C}}}{\partial t} + \overline{u}\frac{{\partial \overline{C}}}{\partial x} + \overline{v}\frac{{\partial \overline{C}}}{\partial y}} \right) = D_{tri - HNF} \frac{{\partial^{2} \overline{C}}}{{\partial y^{2} }} + \frac{{D_{m} K_{T} }}{{T_{S} }}\frac{{\partial^{2} \overline{T}}}{{\partial y^{2} }} - K_{1} \left( {\overline{C} - \overline{C}_{\infty } } \right)^{m} \left( {\frac{{\overline{T}}}{{\overline{T}_{\infty } }}} \right)exp\left( {\frac{{ - E_{a} }}{{k_{tri - HNF} \overline{T}}}} \right)$$

Subject to boundary conditions^39,40^:

At

5 $$t = 0:\overline{T} = \overline{T}_{\infty } ,\overline{C} = \overline{C}_{\infty } ,\overline{u} = 0 = \overline{v}$$

6 $$t \ge 0:\overline{u} = \overline{\overline{{u_{w} }}} = bx,\overline{v} = 0,k_{tri - HNF} \frac{{\partial \overline{T}}}{\partial y} = H_{f} \left( {\overline{T} - \overline{T}_{w} } \right)D_{tri - HNF} \frac{{\partial \overline{C}}}{\partial y} = H_{m} \left( {\overline{C} - \overline{C}_{w} } \right)at \, y = 0$$

7 $$\overline{u} = \overline{\overline{u}}_{e} \to cx,\overline{T} \to \overline{T}_{\infty } ,\overline{C} \to \overline{C}_{\infty } ,at \, y = 0$$

where, $\overline{u }$ and $\overline{v }$ are the velocity constituents, ${\overline{T} }_{\infty }, {\overline{T} }_{w}$ and ${\overline{C} }_{\infty }, {\overline{C} }_{w}$ are the ambient, wall temperature and concertation respectively. Furthermore, ${J}_{0}$ is the Current density, ${M}_{0}$ is the magnetization of permanent magnets, $d$ is the width between magnets and electrodes. Additionally, ${\overline{\overline{u}}}_{e}$, $\rho$, ${c}_{p}$, ${\sigma }^{*}$, ${k}^{*}$, ${k}_{f}$, ${D}_{tri-HNF}$, ${K}_{T}$, ${T}_{S}$, ${K}_{1}$, ${E}_{a}$, ${H}_{f}$ and ${H}_{m}$ are the far field velocity, density, heat capacity, Stefan Boltzmann, mean absorption, thermal conductivity, diffusion, thermal diffusion, chemical reaction, activation energy, energy and mass transmission coefficient, respectively.

Introducing the proper similarity transformation as suggested by^9,41–43^:

8 $$u = \frac{{axf^{\prime}\left( \eta \right)}}{1 - \beta t},v = - \sqrt {\frac{{av_{f} }}{1 - \beta t}} f\left( \eta \right),\eta = y\sqrt {\frac{a}{{v_{f} 1 - \beta t}}} ,\Theta \left( \eta \right) = \frac{{\overline{T} - \overline{T}_{\infty } }}{{\overline{T}_{w} - \overline{T}_{\infty } }},\Phi \left( \eta \right) = \frac{{\overline{C} - \overline{C}_{\infty } }}{{\overline{C}_{w} - \overline{C}_{\infty } }}$$

The dimensionless equations in the view of Eq. (8), becomes

9 $$\frac{{\mu_{tri - HNF} }}{{\mu_{f} }}f^{\prime\prime\prime} + \frac{{\rho_{tri - HNF} }}{{\rho_{f} }}\left( {ff^{\prime\prime} - f^{^{\prime}2} } \right) - A\left( {\frac{\eta }{2}f^{^{\prime\prime}} + f^{\prime} - 1} \right) + \frac{{\rho_{tri - HNF} }}{{\rho_{f} }}M_{r} exp\left( { - \delta \eta } \right) + A^{2} = 0$$

10 $$\left( {\frac{{k_{tri - HNF} }}{{k_{f} }} + Nr} \right)\Theta^{\prime\prime} + \frac{{\left( {\rho c_{p} } \right)_{tri - HNF} }}{{\left( {\rho c_{p} } \right)_{f} }}\Pr \left( {\left( {f^{\prime}\Theta - f\Theta^{\prime}} \right) - \frac{\eta }{2}A\Theta^{\prime}} \right) + \frac{{D_{tri - HNF} }}{{D_{f} }}D_{f} \Psi^{\prime\prime} = 0$$

11 $$\frac{{D_{tri - HNF} }}{{D_{f} }}\Psi^{\prime\prime} + Scf\Psi^{\prime} - \frac{\eta }{2}\Psi^{\prime} - Sc\chi \left( {1 + \omega \Theta } \right)^{m} \exp \left( { - \frac{{E_{A} }}{{\left( {1 + \omega \Theta } \right)}}} \right) + \frac{{k_{tri - HNF} }}{{k_{f} }}Sr\Theta^{\prime\prime} = 0$$

12 $$f^{\prime}\left( 0 \right) = 1, f\left( 0 \right) = 1, \Theta^{\prime}\left( 0 \right) = - \frac{{k_{tri - HNF} }}{{k_{f} }}Bi_{1} \left( {1 - \Theta \left( 0 \right)} \right)\Psi^{^{\prime\prime}} (0) = - \frac{{D_{tri - HNF} }}{{D_{f} }}Bi_{2} (1 - \Psi (0))$$

13 $$f{\prime} (\infty ) = A,\Theta \left( \infty \right) = 0,\Psi (\infty ) = 0$$

where,

**Table:** 

${M}_{r}=\frac{\pi {J}_{0}{M}_{0}}{8{\rho }_{f}}\frac{1-\beta t}{{u}_{w}^{2}}$	Modified Hartman number
$A=\frac{c}{b}$	Stagnation point
$\delta =\frac{\pi }{\sqrt{a/{v}_{f}\left(1-\beta t\right)}}$	Width of magnets and electrodes
$Pr=\frac{{\left(\mu {c}_{p}\right)}_{f}}{{k}_{f}}$	Prandtl number
${D}_{f}=\frac{{D}_{f}{K}_{T}\left({C}_{w}-{C}_{\infty }\right)}{{\nu }_{f}{C}_{S}{c}_{p}\left({T}_{w}-{T}_{\infty }\right)}$	Dufour number
${S}_{r}=\frac{{D}_{f}{K}_{T}\left({T}_{w}-{T}_{\infty }\right)}{\nu {T}_{S}\left({C}_{w}-{C}_{\infty }\right)}$	Soret number
$Sc=\frac{{\nu }_{f}}{{D}_{m}}$	Schmidt number
$Nr=\frac{4{\sigma }^{*}{T}_{\infty }^{3}}{{k}^{*}{k}_{f}}$	Radiative parameter
${E}_{A}=\frac{{E}_{a}}{{k}_{f}{T}_{\infty }}$	Activation energy
$\chi =\frac{{K}_{1}}{b}$	Chemical reaction parameter
$\omega =\frac{{\overline{T} }_{w}-{\overline{T} }_{\infty }}{{\overline{T} }_{\infty }}$	Temperature difference parameter
${Bi}_{1}=\sqrt{\frac{a}{{v}_{f}1-\beta t}}\frac{{H}_{f}}{{k}_{f}}$	Thermal Biot number
${Bi}_{2}=\sqrt{\frac{a}{{v}_{f}\left(1-\beta t\right)}}\frac{{H}_{m}}{{D}_{f}}$	Solutal Biot number

The quantity of engineering concern are the skin friction ${C}_{f}$, Nusselt ${Nu}_{x}$ and Sherwood number ${Sh}_{x}$ are defined as

14 $$C_{f} = \frac{{\tau_{w} }}{{\rho_{tri - HNF} u_{w}^{2} }},Nu_{x} = \frac{{xq_{w} + q_{r} }}{{k_{tri - HNF} \left( {\overline{T}_{w} - \overline{T}_{\infty } } \right)}},and \, Sh_{x} = \frac{{xq_{m} }}{{D_{tri - HNF} \left( {\overline{C}_{w} - \overline{C}_{\infty } } \right)}} \cdot$$

where, ${\tau }_{w}$, ${q}_{w}$ and ${q}_{m}$ are the wall stresses, heat and mass fluxes, respectively. Mathematically

15 $$\tau_{w} = \mu_{tri - HNF} \left. {\left( {\frac{\partial u}{{\partial y}}} \right)} \right|_{y = 0} ,q_{w} = - k_{tri - HNF} \left. {\left( {\frac{\partial T}{{\partial y}}} \right)} \right|_{y = 0} , q_{m} = - D_{tri - HNF} \left. {\left( {\frac{\partial C}{{\partial y}}} \right)} \right|_{y = 0}$$

In dimensionless form, the physical quantities becomes

16 $${\mathrm{Re}}_{x}^{(1/2)} C_{f} = \frac{{\mu_{tri - HNF} }}{{\mu_{f} }}f^{^{\prime\prime}} (0),{\mathrm{Re}}_{x}^{( - 1/2)} Nu_{x} = - (\frac{{k_{tri - HNF} }}{{k_{f} }} + Nr)\Theta{\prime} (0),Sh_{x} = - \frac{{D_{tri - HNF} }}{{D_{f} }}\Psi^{\prime}\left( 0 \right)$$

where, ${Re}_{x}^{1/2}=\frac{{u}_{x}x}{{\nu }_{f}}$ is the local Reynold number.

### Thermo-physical and rheological features of tri-HNF

The mathematical expressions for density of mono, hybrid and tri-HNF are defined as^44,45^:

17 $$\frac{{\rho_{nf} }}{{\rho_{f} }} = \left( {1 - \phi_{1} } \right) + \phi_{1} \frac{{\rho_{bf} }}{{\rho_{f} }}$$

18 $$\frac{{\rho_{HNF} }}{{\rho_{nf} }} = \left[ {\left\{ {\left( {1 - \phi_{1} } \right) + \frac{{\phi_{1} \rho_{np1} }}{{\rho_{nf} }}} \right\}\left( {1 - \phi_{2} } \right) + \frac{{\phi_{2} \rho_{np2} }}{{\rho_{nf} }}} \right]$$

19 $$\rho_{tri - HNF} = \left( {1 - \phi_{3} } \right)\left[ {\left\{ {\left( {1 - \phi_{1} } \right)\rho_{bf} + \phi_{1} \rho_{np1} } \right\}\left( {1 - \phi_{2} } \right) + \phi_{2} \rho_{np2} } \right] + \phi_{3} \rho_{np3}$$

And

20 $$\frac{{\rho_{tri - HNF} }}{{\rho_{f} }} = \left( {1 - \phi_{3} } \right)\left[ {\left\{ {\left( {1 - \phi_{1} } \right) + \phi_{1} \frac{{\rho_{np1} }}{{\rho_{f} }}} \right\}\left( {1 - \phi_{2} } \right) + \phi_{2} \frac{{\rho_{np2} }}{{\rho_{f} }}} \right] + \phi_{3} \frac{{\rho_{np3} }}{{\rho_{f} }}$$

The mathematical expressions for viscosity of mono, hybrid and tri-HNF are defined as^46,47^:

21 $$\mu_{nf} = \frac{{\mu_{f} }}{{\left( {1 - \phi_{1} } \right)^{2.5} }}$$

22 $$\mu_{HNF} = \frac{{\mu_{f} }}{{\left( {1 - \phi_{1} } \right)^{2.5} \left( {1 - \phi_{2} } \right)^{2.5} }}$$

23 $$\mu_{tri - HNF} = \frac{{\mu_{f} }}{{\left( {1 - \phi_{1} } \right)^{2.5} \left( {1 - \phi_{2} } \right)^{2.5} \left( {1 - \phi_{3} } \right)^{2.5} }}$$

The mathematical expressions for heat capacity of mono, hybrid and tri-HNF are defined as^46^:

24 $$(C_{p} )_{nf} = \left( {1 - \phi_{1} } \right)(C_{p} )_{bf} + \phi_{1} \left( {C_{p} } \right)_{p1}$$

25 $$(C_{p} )_{HNF} = \left\{ {\left( {1 - \phi_{1} } \right)(C_{p} )_{bf} + \phi_{1} \left( {C_{p} } \right)_{np1} } \right\}\left( {1 - \phi_{2} } \right) + \phi_{1} \left( {C_{p} } \right)_{np2}$$

26 $$(C_{p} )_{tri - HNF} = \left[ {\left\{ {\left( {1 - \varphi_{1} } \right)(C_{p} )_{f} + \varphi_{1} \left( {C_{p} } \right)_{p1} } \right\}\left( {1 - \varphi_{2} } \right) + \varphi_{1} \left( {C_{p} } \right)_{p2} } \right]\left( {1 - \varphi_{3} } \right) + \varphi_{3} \left( {C_{p} } \right)_{p3}$$

And

27 $$\frac{{(C_{p} )_{tri - HNF} }}{{(C_{p} )_{f} }} = \left[ {\left\{ {\left( {1 - \phi_{1} } \right) + \phi_{1} \frac{{\left( {C_{p} } \right)_{np1} }}{{(C_{p} )_{f} }}} \right\}\left( {1 - \phi_{2} } \right) + \frac{{\phi_{1} \left( {C_{p} } \right)_{np2} }}{{(C_{p} )_{f} }}} \right]\left( {1 - \phi_{3} } \right) + \phi_{3} \frac{{\left( {C_{p} } \right)_{np3} }}{{(C_{p} )_{f} }}$$

The mathematical expressions for thermal conductivity of mono, hybrid and tri-HNF are defined as^48^

28 $$\frac{{k_{nf} }}{{k_{f} }} = \frac{{\left( {k_{np1} + 2k_{bf} } \right) - \left( {k_{bf} - k_{np1} } \right)2\phi_{1} }}{{\left( {k_{np1} + 2k_{bf} } \right) + \left( {k_{bf} - k_{np1} } \right)\phi_{1} }}$$

29 $$\frac{{k_{HNF} }}{{k_{nf} }} = \frac{{\left( {k_{np2} + 2k_{nf} } \right) - \left( {k_{nf} - k_{np2} } \right)2\phi_{2} }}{{\left( {k_{np2} + 2k_{nf} } \right) + \left( {k_{nf} - k_{np2} } \right)\phi_{2} }}$$

30 $$\frac{{k_{tri - HNF} }}{{k_{HNF} }} = \frac{{\left( {k_{np3} + 2k_{HNF} } \right) - \left( {k_{HNF} - k_{np3} } \right)2\phi_{3} }}{{\left( {k_{pn3} + 2k_{HNF} } \right) + \left( {k_{HNF} - k_{np3} } \right)\phi_{3} }}$$

And

31 $$\frac{{k_{tri - HNF} }}{{k_{bf} }} = \left[ {\frac{{\left( {k_{np3} + 2k_{HNF} } \right) - \left( {k_{HNF} - k_{np3} } \right)2\phi_{3} }}{{\left( {k_{np3} + 2k_{HNF} } \right) + \left( {k_{HNF} - k_{np3} } \right)\phi_{3} }}} \right]\frac{{k_{HNF} }}{{k_{bf} }}$$

The mathematical expressions for nanomaterial diffusion of mono, hybrid and tri-HNF are defined as^48^

32 $$\frac{{D_{nf} }}{{D_{f} }} = \left( {1 - \phi_{1} } \right)$$

33 $$\frac{{D_{HNF} }}{{D_{nf} }} = \left( {1 - \left( {\phi_{1} + \phi_{2} } \right)} \right)$$

34 $$\frac{{D_{tri - HNF} }}{{D_{HNF} }} = \left( {1 - \left( {\phi_{1} + \phi_{2} } \right)} \right)$$

In above expressions, the symbols ${\phi }_{1}$, ${\phi }_{2}$, and ${\phi }_{3}$ showing the nanomaterials load. In Table 1 the thermo-chemo-physical features of water and three nanomaterials are given.

**Table 1:** Thermo-chemo-physical characteristic of water and three nanomaterials^49–51^.

Thermo-physical characteristic	$$\rho$$$$\left[Kg{m}^{-3}\right]$$	$${c}_{p}\left[J{k}^{-1}{g}^{-1}{K}^{-1}\right]$$	$$k\left[W{m}^{-1}{K}^{-1}\right]$$
Water ${H}_{2}O$ (base liquid)	997.1	4179	0.613
Aluminum Oxide $A{l}_{2}{O}_{3}$ (${\phi }_{1}$)	3970	765	40
Copper Oxide $CuO$ (${\phi }_{2}$)	6320	5315	76.5
Titanium Dioxide $Ti{O}_{2}$ (${\phi }_{3}$)	4250	686.2	8.9538

Numerical framework.

The procedure for resolving this BLF problem is explained in this section. The fundamental concept behind RK-4 fourth-order technique is not satisfied by the dimensional partial differential form of the governing Eq. (2)-Eq. (4). First, we create non-dimensional ODEs Eq. (9)-(11) with boundary conditions (BCs) Eq. (12) and (13) by applying the proper transformation Eq. (8). Seventh order equations with seven BCs are produced by non-dimensional equations. Initial conditions are obtained from BCs using the shooting approximation. By calculating the difference between two computations, which guides the step size modification, the correctness of this method is assessed. Appropriate initial estimations are crucial to the shooting method’s successful use. These starting values have a significant impact on the overall result. Iterations continue with a set step size of 0.001 until the answer converges within a predetermined error margin.

Introducing new variables

35 $$Z_{1} = f,Z_{2} = f^{\prime},Z_{2} = f^{\prime\prime},Z^{\prime}_{3} = f^{\prime\prime\prime},Z_{4} = \Theta ,Z_{5} = \Theta^{\prime},Z^{\prime}_{5} = \Theta^{\prime\prime},Z_{6} = \Psi ,Z_{7} = \Psi^{\prime},Z^{\prime}_{7} = \Psi^{\prime\prime}$$

36 $${{\rm Z}}_{3}{\prime} = \frac{1}{{\frac{{\mu_{tri - HNF} }}{{\mu_{f} }}}}\left( { - \frac{{\rho_{tri - HNF} }}{{\rho_{f} }}\left( {{{\rm Z}}_{1} {{\rm Z}}_{3} - {{\rm Z}}_{2}^{2} } \right) + A\left( {\frac{\eta }{2}{{\rm Z}}_{3} + {{\rm Z}}_{2} - 1} \right) - \frac{{\rho_{tri - HNF} }}{{\rho_{f} }}M_{r} exp\left( { - \delta \eta } \right) - A^{2} } \right)$$

37 $${{\rm Z}}_{5}{\prime} = - \frac{1}{{\left( {\frac{{k_{tri - HNF} }}{{k_{f} }} + Nr} \right)}}\left( {\frac{{\left( {\rho c_{p} } \right)_{tri - HNF} }}{{\left( {\rho c_{p} } \right)_{f} }}Pr\left( {\left( {{{\rm Z}}_{2} {{\rm Z}}_{4} - {{\rm Z}}_{1} {{\rm Z}}_{5} } \right) - \frac{\eta }{2}A{{\rm Z}}_{5} } \right) + \frac{{D_{tri - HNF} }}{{D_{f} }}D_{f} {{\rm Z}}_{7}{\prime} } \right)$$

38 $${{\rm Z}}_{7}{\prime} = - \frac{1}{{\frac{{D_{tri - HNF} }}{{D_{f} }}}}\left( {Sc{{\rm Z}}_{1} {{\rm Z}}_{7} - \frac{\eta }{2}{{\rm Z}}_{7} - Sc\chi \left( {1 + \omega {{\rm Z}}_{4} } \right)^{m} exp\left( { - \frac{{E_{A} }}{{\left( {1 + \omega {{\rm Z}}_{4} } \right)}}} \right) + \frac{{k_{tri - HNF} }}{{k_{f} }}Sr{{\rm Z}}_{5}{\prime} } \right)$$

39 $${\rm Z}_{2} \left( 0 \right) = 1, {\rm Z}_{1} \left( 0 \right) = \lambda_{1} , {\rm Z}_{5} \left( 0 \right) = - \lambda_{2} \left( {\frac{{k_{tri - HNF} }}{{k_{f} }}Bi_{1} \left( {1 - {\rm Z}_{4} \left( 0 \right)} \right)} \right),{\rm Z}_{7} \left( 0 \right) = - \lambda_{3} \left( {\frac{{D_{tri - HNF} }}{{D_{f} }}Bi_{2} \left( {1 - {\rm Z}_{6} \left( 0 \right)} \right)} \right)$$

40 $${\rm Z}_{2} \left( \infty \right) = A,{\rm Z}_{4} \left( \infty \right) = 0,{\rm Z}_{6} \left( \infty \right) = 0.$$

where ${\lambda }_{1}$, ${\lambda }_{3}$, and ${\lambda }_{3}$ are unknown which are computed using the Newton procedure from additional BCs. A built-in mathematical program in MATLAB 2019b is used to carry out this complete process. The current model is validated with previous models in a limiting scenarios, setting ${M}_{r}=0$ is shown in Table 2.

**Table 2:** Model validation with previous models.

$A$	Hayat et al^52^.	Mustafa et al^53^.	Shafiq et al^54^.	Current findings
0.1	$-$ 0.96802	$-$ 0.9694	$-$ 0.96803	$-$ 0.9689
0.2	$-$ 0.91692	$-$ 0.9181	$-$ 0.91700	$-$ 0.9169
0.5	$-$ 0.66722	$-$ 0.6673	$-$ 0.66749	$-$ 0.6669
2.0	2.0175	2.0175	2.0175	2.0174
3.0	4.7291	4.7293	4.7292	4.7291

## Results and discussion

In this segment the achieved numerical results via RK-4 are visualized and discussed in detial for range of parameters influencing the flow dynamics, temeprature and concentration of tri-HNF. The tri-HNF is prepared by dispersing three nanomaterials $A{l}_{2}{O}_{3}$, $CuO$ and $Ti{O}_{2}$ of equal load of 5% in water. The analysis are done by varaiying one parameter at a time, while other parameters are fixed such as $Pr=6.2$

**Fig. 2:**
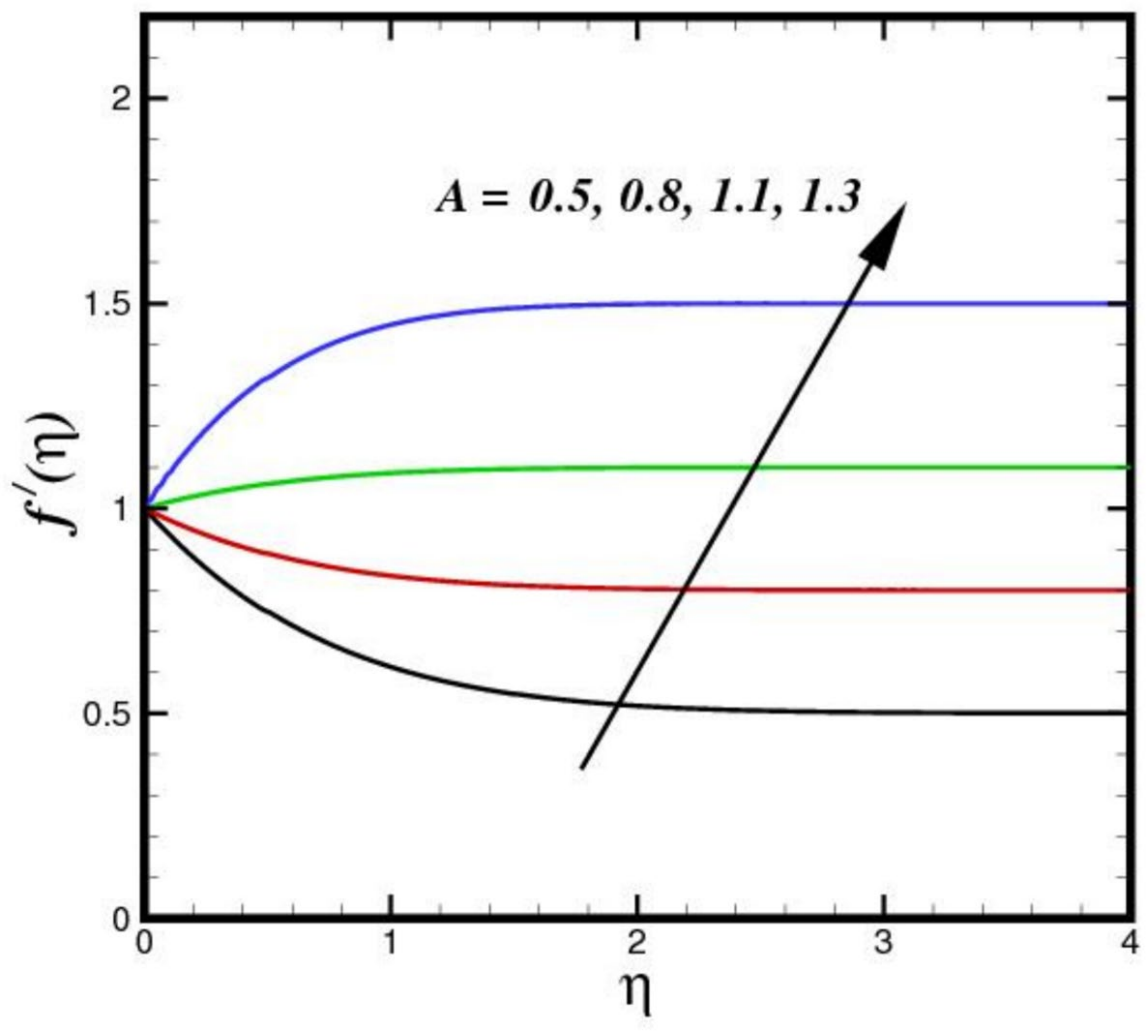
Amplification of $A$ verses ${f}{\prime}\left(\eta \right).$.

**Fig. 3:**
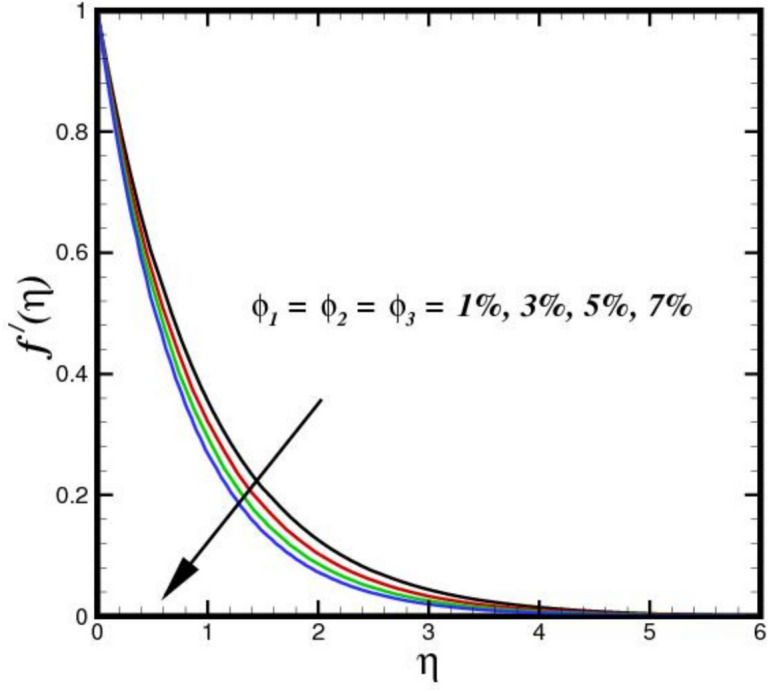
Amplification of ${\phi }_{1}$, ${\phi }_{2}$ and ${\phi }_{3}$ verses ${f}{\prime}\left(\eta \right).$.

**Fig. 4:**
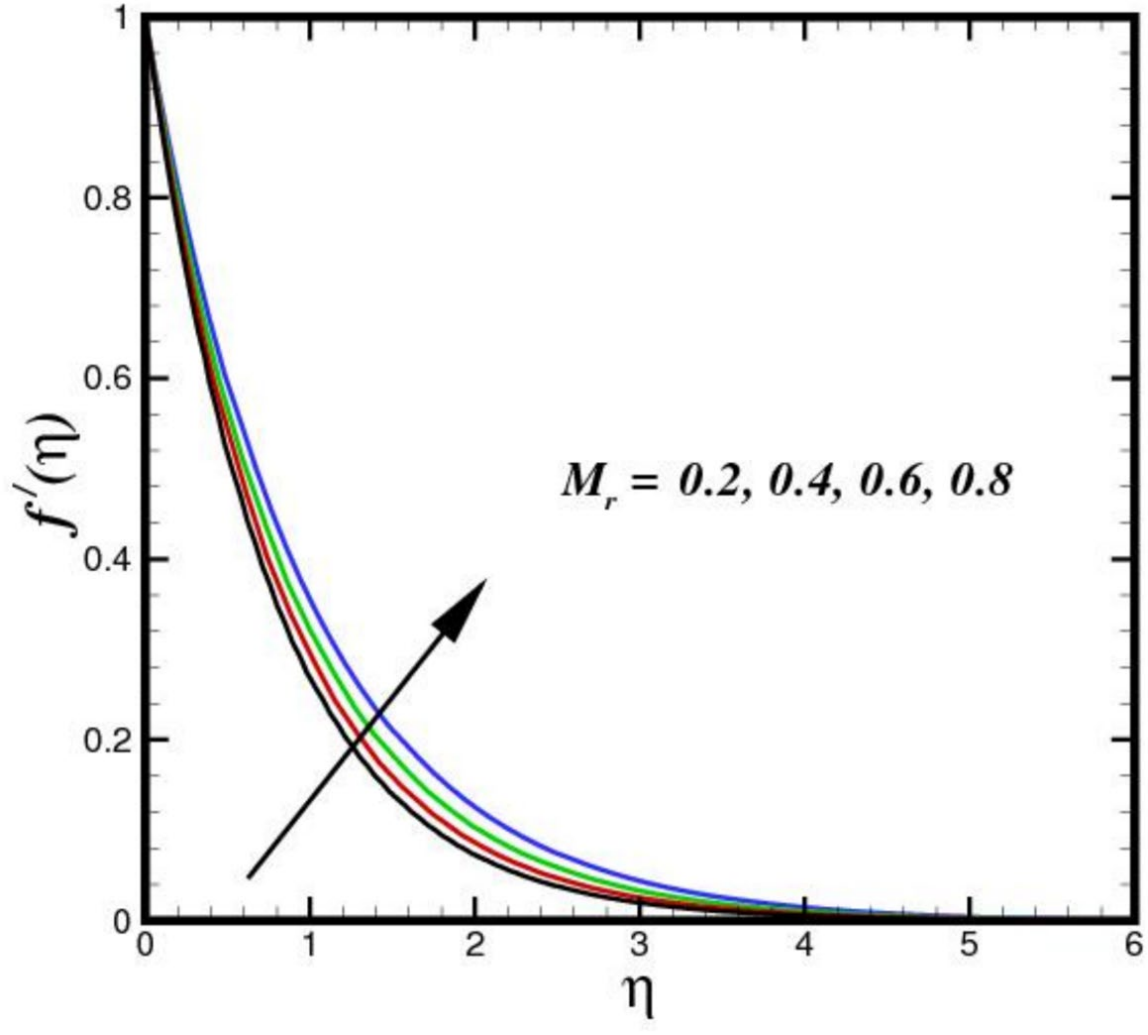
Amplification of ${M}_{r}$ verses ${f}{\prime}\left(\eta \right).$.

**Fig. 5:**
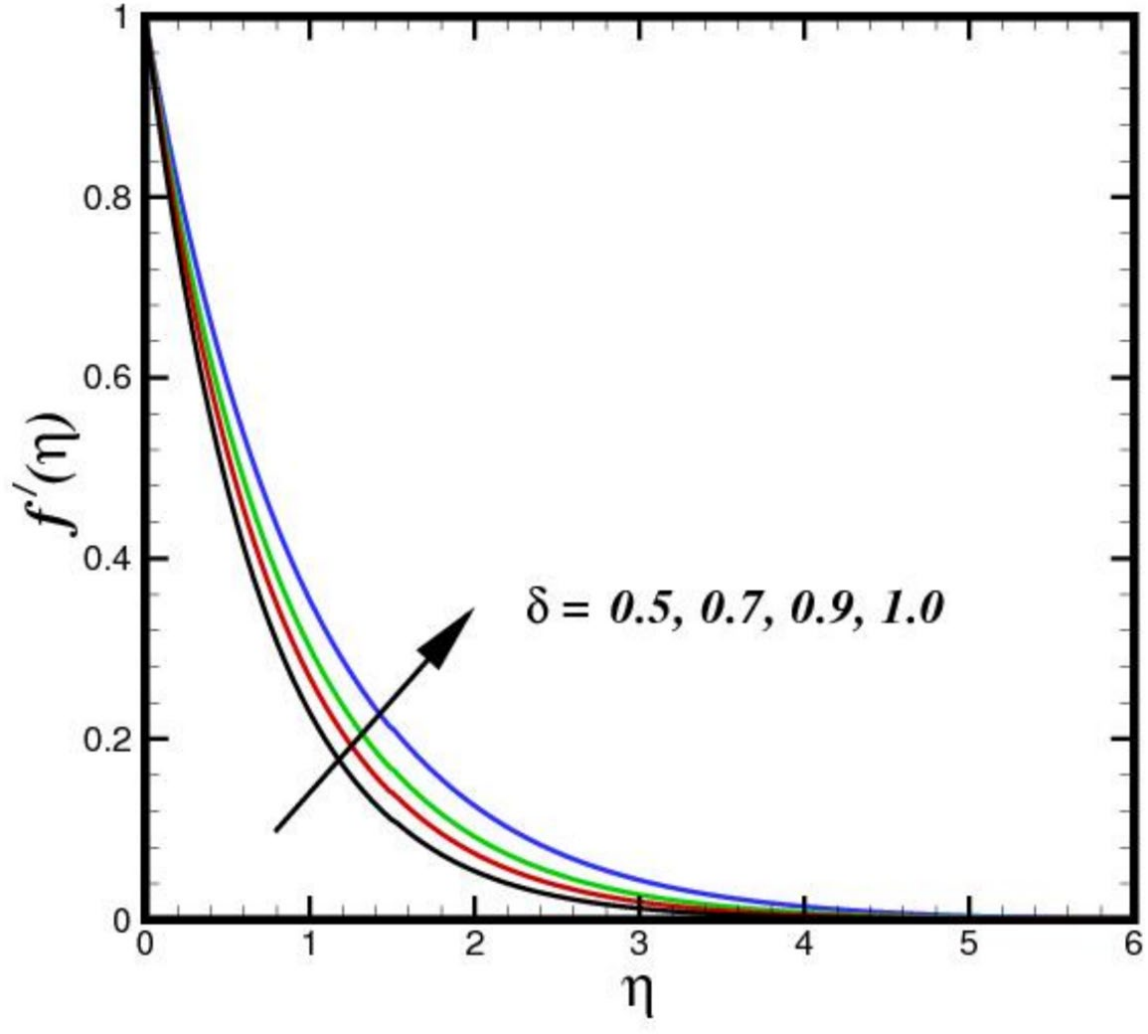
Amplification of $\delta$ verses ${f}{\prime}\left(\eta \right).$.

The stagnation point parameter $A$ influences velocity by altering the flow due to pressure gradient near the stagnation region. A higher $A$ increases the adverse pressure gradient, aecelerating the flow more sharply as it approaches the stagnation point as shown in Fig. 2. This results in a thicker BL and reduced velocity ${f}{\prime}\left(\eta \right)$ magnitudes near the surface, as more kinetic energy is converted into pressure, emphasizing stagnation effects. Conversely, a lower parameter weakens the gradient, allowing faster flow velocities closer to the point. Figure 3 illustrates the effect of three nanomaterials of a 5% load on the flow profile ${f}{\prime}\left(\eta \right)$. A tri-HNF across a Riga plate has less impact on ${f}{\prime}\left(\eta \right)$ due to three nanomaterials because of enhanced contacts between nanoparticles and a larger effective viscosity, which reduces fluid motion ${f}{\prime}\left(\eta \right)$. The improved thermal conductivity and thickening of the BL minimize velocity as the concentration of nanoparticles increases, resulting in a flatter ${f}{\prime}\left(\eta \right)$. Furthermore, the flow dynamics are further limited by Lorentz forces from the Riga plate, which increases the decelerating effect of nanomaterials. Therefore, a decreasing flow profile is the outcome of the combined influence of electromagnetic phenomena and nanoparticle loading. Figure 4 displays the ${f}{\prime}\left(\eta \right)$ contours for a range of modified Hartman numbers ${M}_{r}$, at predetermined values for the other parameters. It was found that when ${M}_{r}$ increases, consequently increases ${f}{\prime}\left(\eta \right)$, which is associated with the horizontal velocity. This indicates that the surface-parallel Lorentz force contributes to support the flow in the horizontal direction. Additionally, as the magnetic field strength increases, the wall velocity gradient widens and the BL thickness decreases. The profile ${f}{\prime}\left(\eta \right)$ overshoot is seen for larger values of ${M}_{r}$, indicating that the fluid velocity near the plate is greater than the free stream velocity when Lorentz force are stronger enough. The velocity progression in the BL regime transverse to the sheet with an increase in $\delta$ is depicted in Fig. 5. The velocity increases as $\delta$ increases, meaning that the flow accelerates and the thickness of the momentum BL decreases. Since $\delta$ is directly proportional to the magnitization of the Riga surface. Therefore, the resulting magnetic body force in line with the sensor surface and is not retarding way. This promotes velocities and aids in momentum growth. Thus, when magnetization disappears, i.e., ${M}_{r}=0$ and consequently $\delta =0$, velocity is reduced, resulting in the highest width of the momentum BL.

**Fig. 6:**
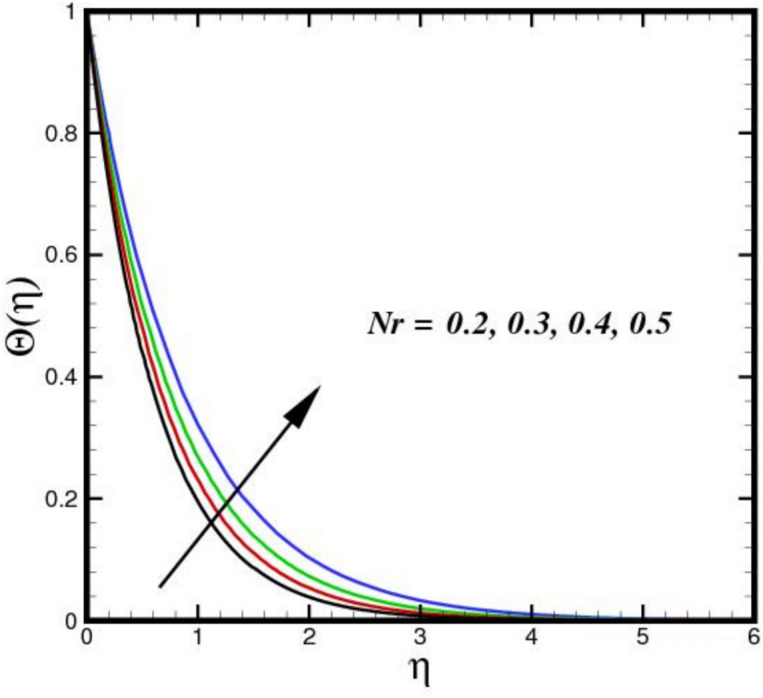
Amplification of $Nr$ verses $\Theta \left(\eta \right).$.

**Fig. 7:**
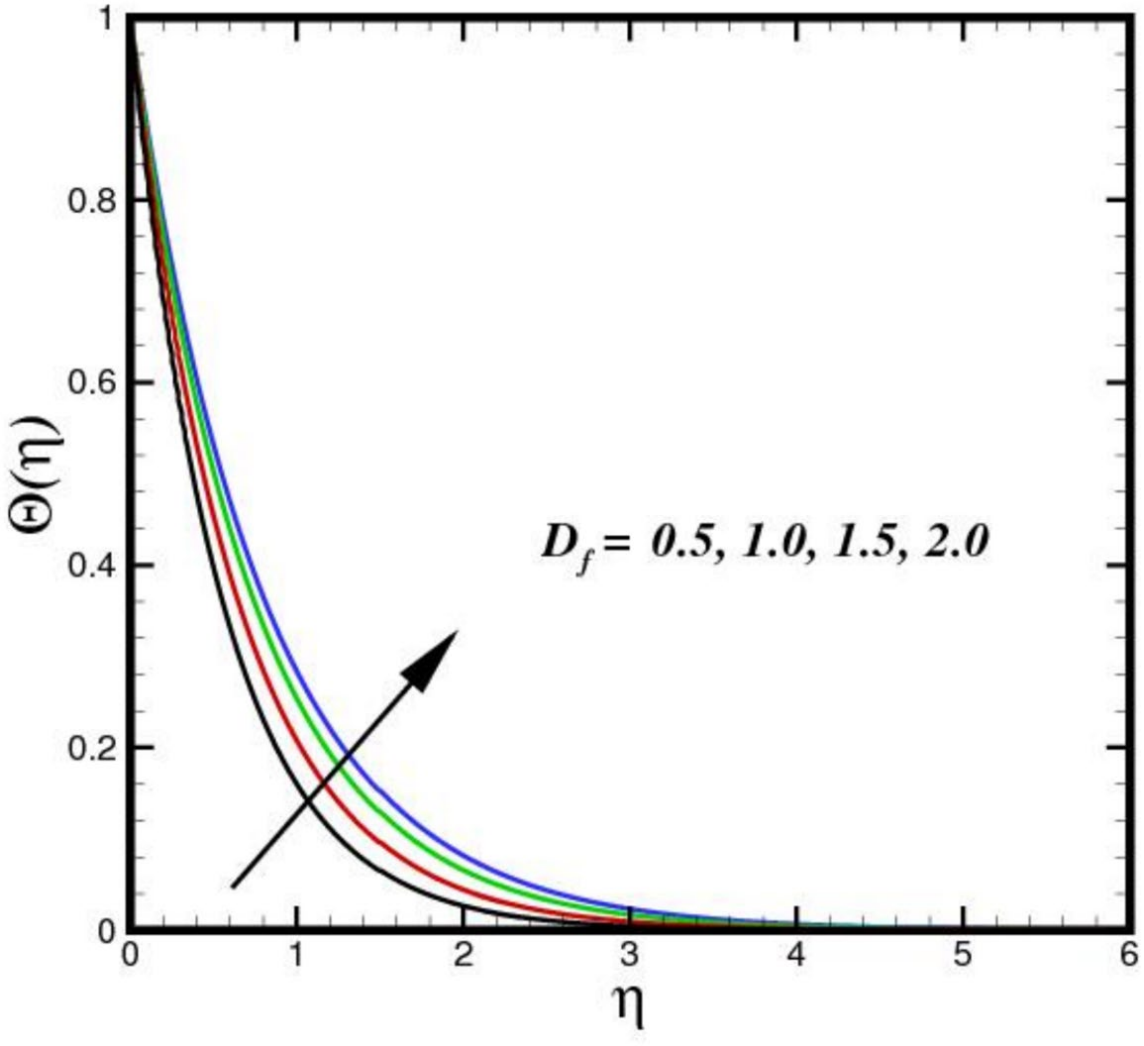
Amplification of ${D}_{f}$ verses $\Theta \left(\eta \right).$.

**Fig. 8:**
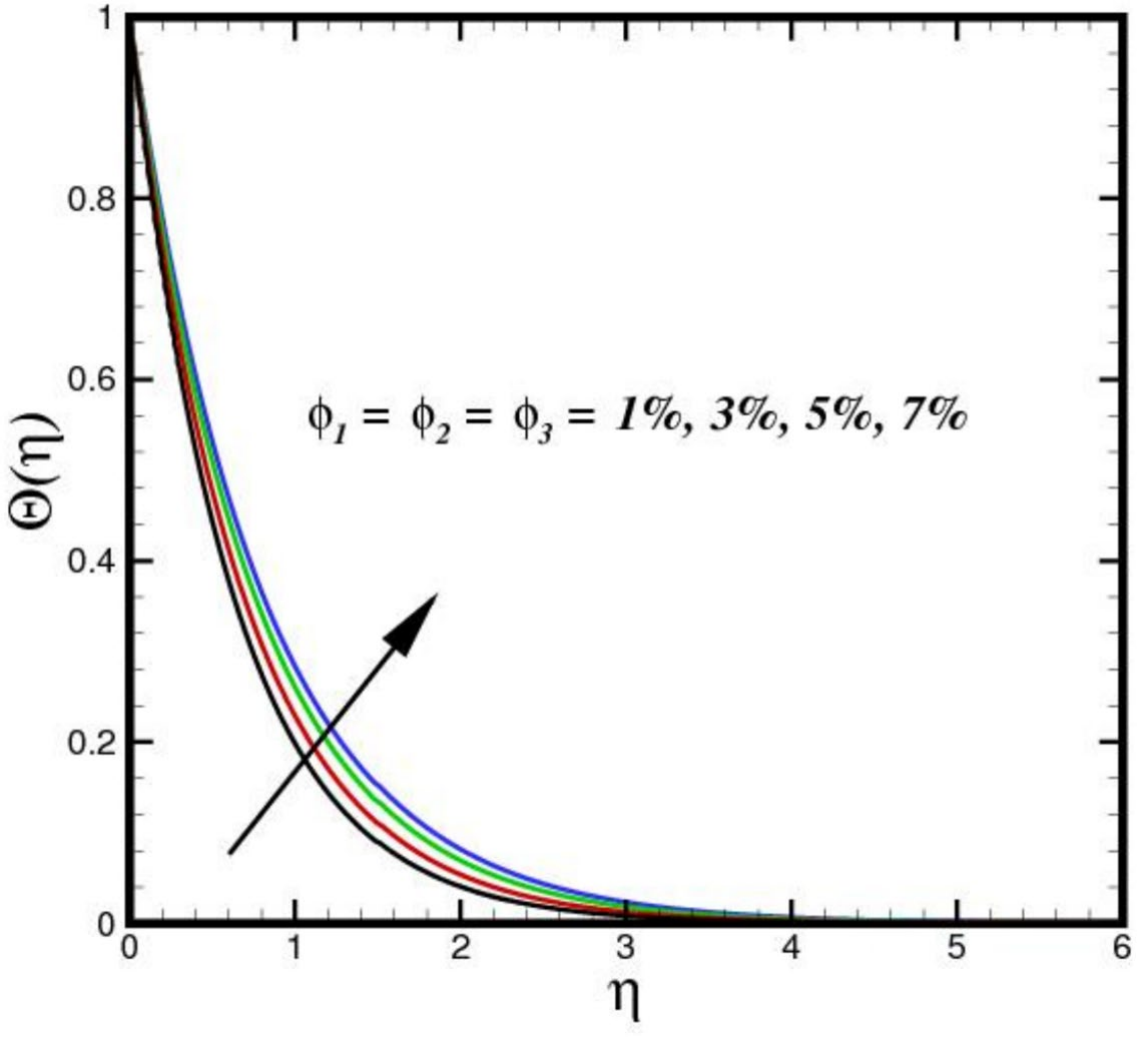
Amplification of ${\phi }_{1}$, ${\phi }_{2}$ and ${\phi }_{3}$ verses $\Theta \left(\eta \right).$.

**Fig. 9:**
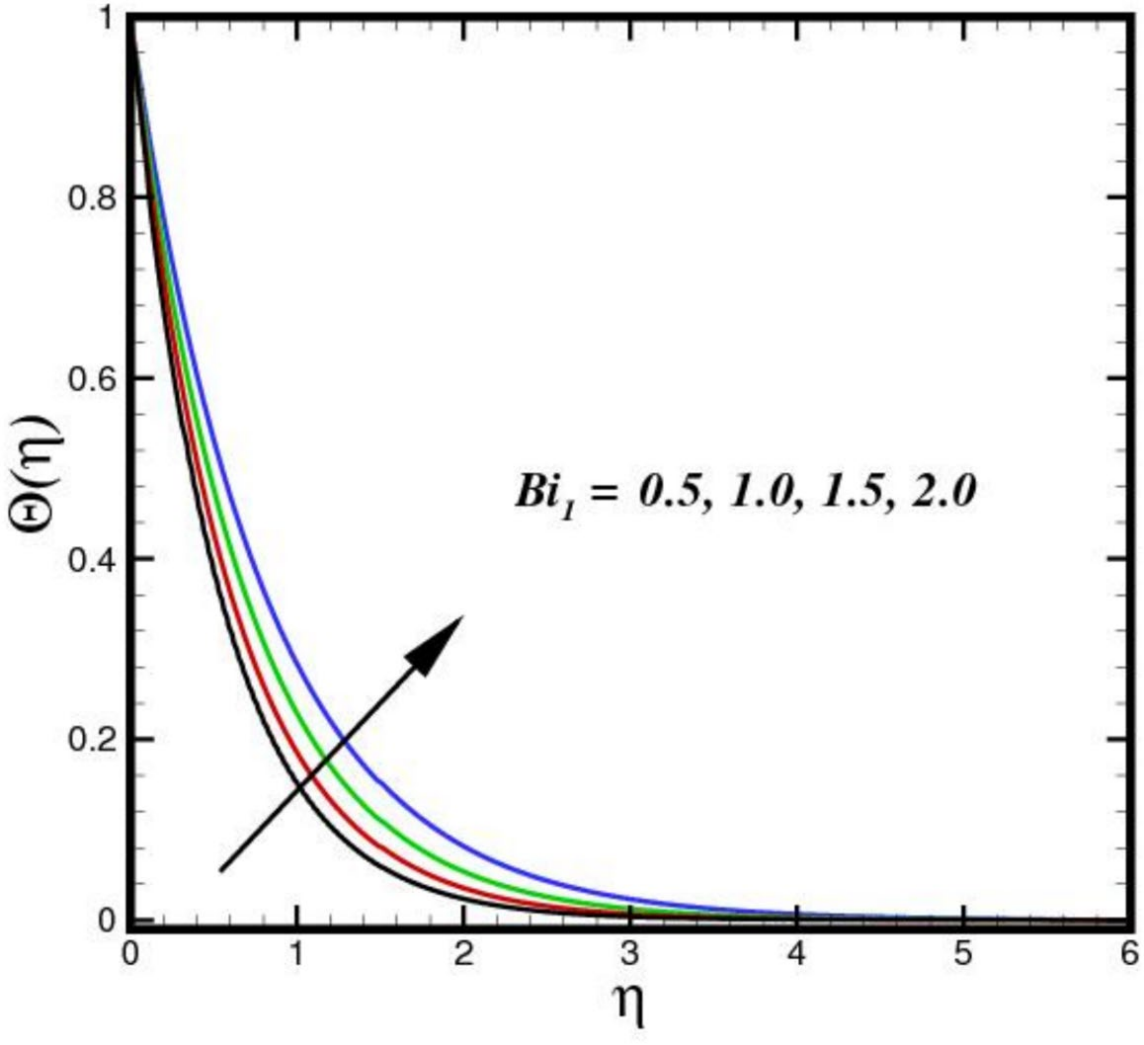
Amplification of ${Bi}_{1}$ verses $\Theta \left(\eta \right).$.

The impact of thermal radiation parameter $Nr$ on the thermal profile $\Theta \left(\eta \right)$ is seen in Fig. 6. The figure clearly shows that temperature and $Nr$ have a direct relationship, meaning that temperature rises as $Nr$ increases in magnitude. Physically, because of its electrical and magnetic properties, the Riga plate surface endures the greatest thermal diffusions as the values of $Nr$ increases. Thus, an expanded thermal distribution results from an increase in the $Nr.$ In Fig. 7, the consequence of ${D}_{f}$ on $\Theta \left(\eta \right)$ is displayed. The thermal flux induced by a concentration gradient is referred to as the Dufour effect. The thermal profile is strong, when ${D}_{f}$ is present and respond adversely, when it is not present. The thickness of the thermal BL increases considerably as ${D}_{f}$ increases, suggesting that the BLF is more active. The effect of three nanomaterials load on $\Theta \left(\eta \right)$ of a tri-HNF flow over a Riga plate is depicted in Fig. 8. An increase in $\Theta \left(\eta \right)$ due to higher thermal conductivity and energy absorption from the nanoparticles. The synergistic interaction of the three nanomaterials improves heat transfer efficiency, raising fluid temperatures. Additionally, the Lorentz forces created by the Riga plate induce Joule heating, further amplifying thermal effects. As a result, the combined influence of nanoparticle properties and electromagnetic heating leads to a higher $\Theta \left(\eta \right)$ in the BL. Figure 9 illustrates the impact of the Biot number $B{i}_{1}$ on $\Theta \left(\eta \right)$. A higher $B{i}_{1}$ improves convective heat transport at the surface, which raises $\Theta \left(\eta \right)$. In order to promote more effective heat transfer between the fluid and surroundings, a higher $B{i}_{1}$ denotes decreased thermal resistance in the BL. This causes a more noticeable thermal BL by raising the overall $\Theta \left(\eta \right)$ and creating a steeper temperature differential close to the surface.

**Fig. 10:**
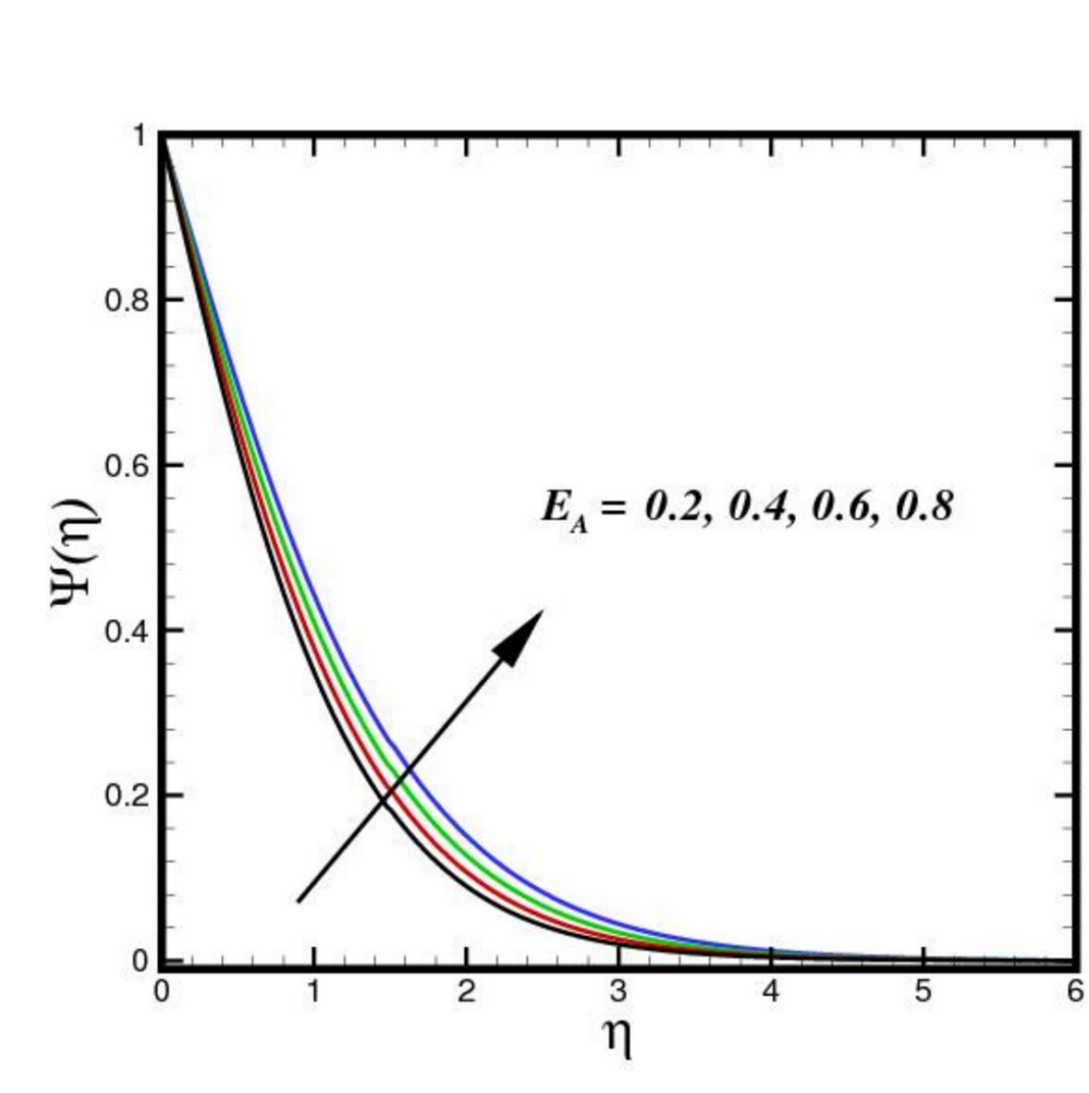
Amplification of ${E}_{A}$ verses $\Psi \left(\eta \right).$.

**Fig. 11:**
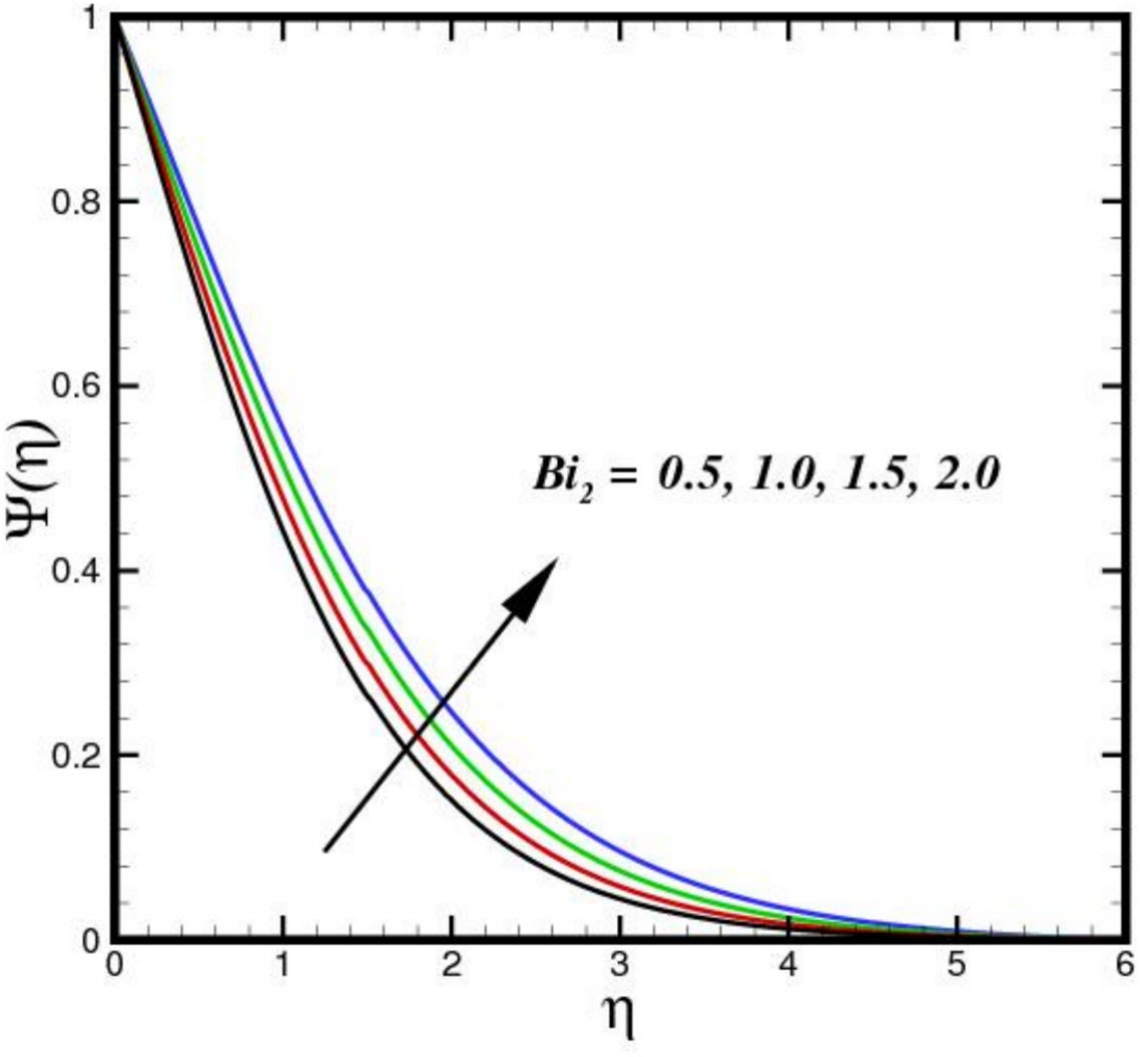
Amplification of ${Bi}_{2}$ verses $\Psi \left(\eta \right).$.

**Fig. 12:**
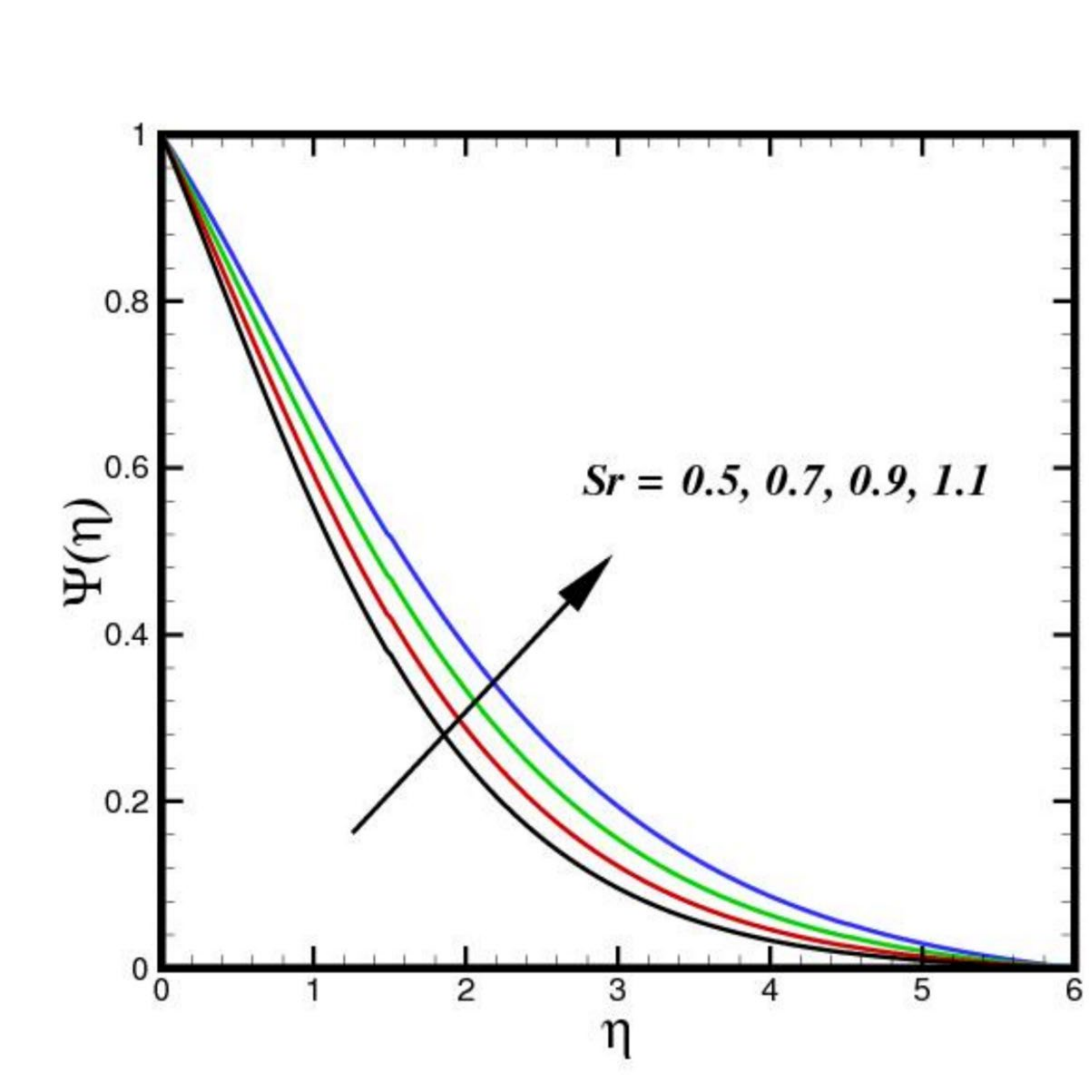
Amplification of $Sr$ verses $\Psi \left(\eta \right).$.

**Fig. 13:**
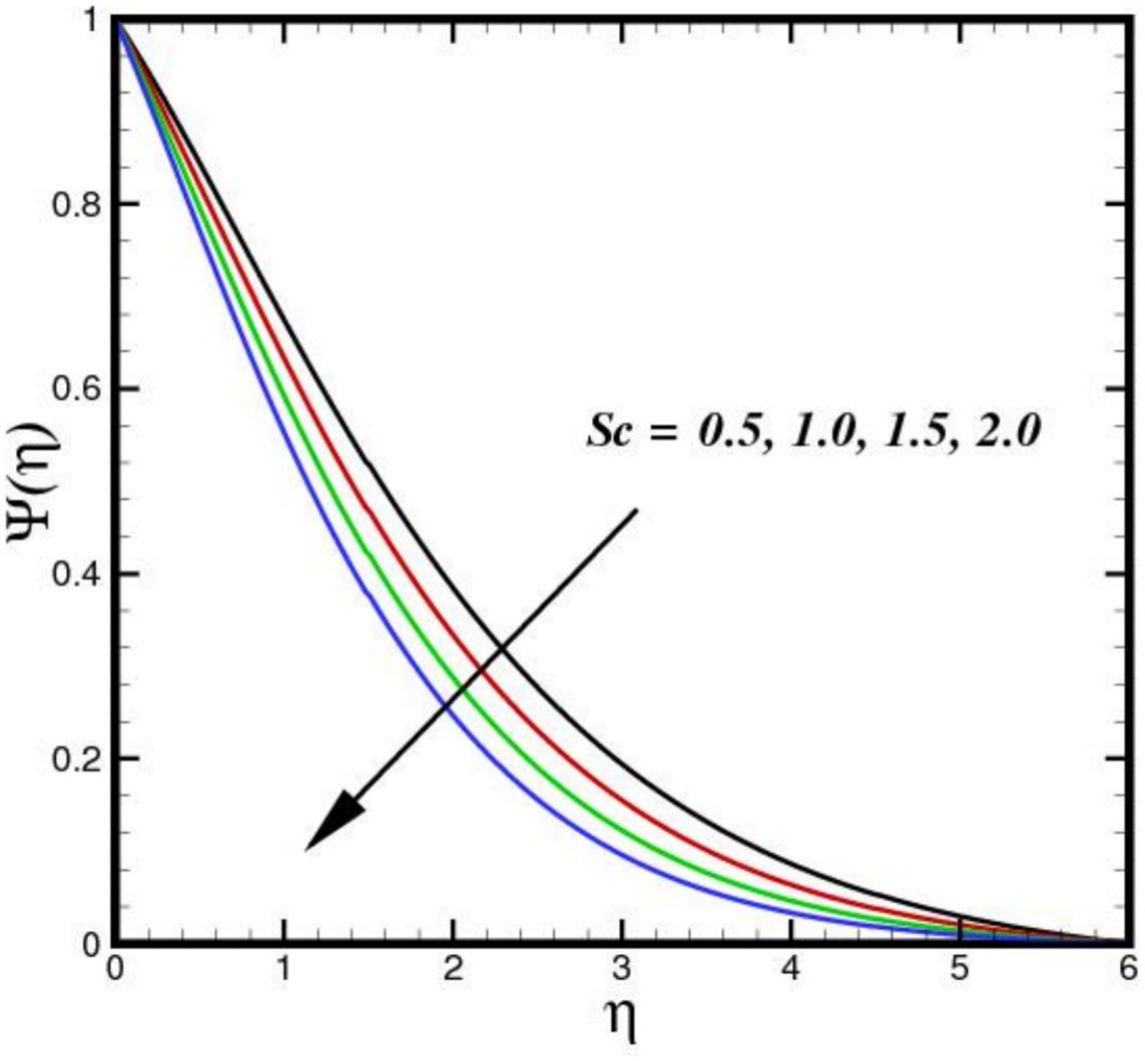
Amplification of $Sc$ verses $\Psi \left(\eta \right).$.

**Fig. 14:**
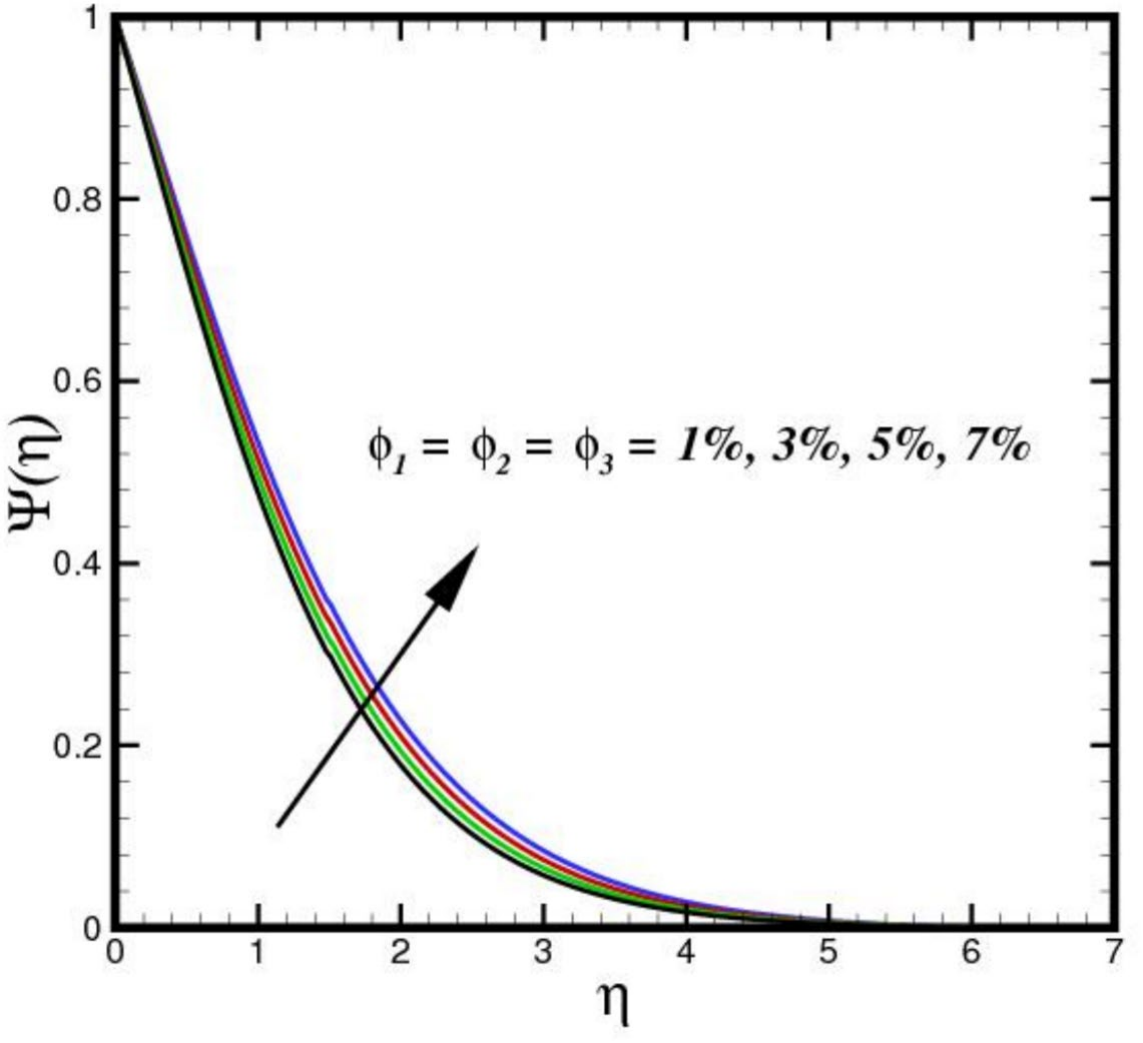
Amplification of ${\phi }_{1}$, ${\phi }_{2}$ and ${\phi }_{3}$ verses $\Psi \left(\eta \right).$.

The outcome of the activation energy ${E}_{A}$ on concentration outline $\Psi (\eta )$ is shown in Fig. 10. The concentration profile increases as the ${E}_{A}$ rises. The rapidity of chemical reactions is related to ${E}_{A}$. It calculates the energy needed for molecules to move through a fluid. Reactions or processes that depend on temperature are linked to activation energy. The fluid molecules require more energy to move or reorganize themselves when ${E}_{A}$ is higher. As a result, more energy is required in the combination solution to produce the same amount of molecular motion. Changes in molecular diffusion speeds are linked to an increase in ${E}_{A}$. Slower diffusion brought on by higher ${E}_{A}$ may alter the fluid solute concentration profile. The influence of $B{i}_{2}$ on $\Psi (\eta )$ is shown in Fig. 11. The ratio of internal species transfer resistance in relation to exterior transfer impedance is determined by $B{i}_{2}$. By enhancing mass transport at the surface, a higher $B{i}_{2}$ improves the concentration profile. Better contact across the fluid and the surrounding medium is made possible by a higher $B{i}_{2}$, which lowers the resistance to mass diffusion. As a result, the concentration BL becomes more noticeable due to a steeper concentration gradient close to the boundary and an overall increase in solute distribution. The effect of the Soret number $Sr$ on $\Psi (\eta )$ is seen in Fig. 12. By enhancing thermal diffusion, where variations in temperature promote mass transfer. A rise in $Sr$ improves the concentration profile. Physically, more solute movement from hotter to cooler regions results from a higher $Sr$, which denotes an increased impact of thermal diffusion over molecular diffusion. As a result, the concentration gradient becomes steeper and the redistribution of nanoparticles within the BL becomes more noticeable. The outcomes of $Sc$ on concentration outline is shown in Fig. 13. The concentration outline shrinks as the $Sc$ surges. The relative significance of mass diffusion and momentum diffusion in a fluid flow is described by the Schmitt number $Sc$. In a tri-HNF flow past a Riga plate, the momentum diffusion is more prevalent than mass diffusion when $Sc$ is higher. The effects of three nanomaterials volume percentage on $\Psi (\eta )$ are shown in Fig. 14. Because of higher mass diffusion and nanoparticle interactions, the addition of three nanomaterials improves the concentration profile of tri-HNF. When three nanoparticles interact, they enhance thermophoresis and Brownian motion, which encourages more particle migration and dispersion in the fluid. As a result, the solute concentration rises and the concentration BL becomes more noticeable.

Response surface methodology (RSM)

RSM a robust statistical and mathematical framework for modeling and optimizing the interaction between a response variable and several input variables is widely applied in science, engineering, and industrial engineering. RSM can be used to investigate and comprehend how different elements affect a fluid or material shear rate, and in the end, determine the ideal circumstances that optimize or reduce the shear rate in accordance with particular goals. In order to effectively investigate an input parameter space and develop a prediction model for the response parameter the shear rate, heat and mass fluxes, RSM entails the development and evaluation of experiments. Three primary processes are usually involved in the process: the Data Collection, Modeling, and Experimental Design^33,55^. At this point a number of variables pertaining to the suggested flow phenomenon are taken into account while analyzing the shear rate and heat transfer rate responses.

Optimization of wall stresses, nusselt and sherwood number.

A face-centered composite structure was used to examine the relationship among the contributing variables and the response indicator, wall stresses, temperature and concentration gradients. The three components and their levels are shown in Table 3. The quadratic models in Eq. (41), (42) and (43) involves three linear, square, and interaction variables, presents. A built-in "central composite design" (CCD) with several layers, such as linear, square, and interaction terms, is suggested for modeling the quadratic order model. To separate these ranges into lower, middle, and highest values, the face-centered technique is applied.

41 $$Re_{x}^{{1/2}} C_{f} = {\mathbbm{a}}_{0} + {\mathbbm{a}}_{1} {\mathbb{A}} + {\mathbbm{a}}_{2} {\mathbb{B}} + {\mathbbm{a}}_{3} {\mathbb{C}} + {\mathbbm{a}}_{{11}} {\mathbb{A}}^{2} + {\mathbbm{a}}_{{22}} {\mathbb{B}}^{2} + {\mathbbm{a}}_{{33}} {\mathbb{C}}^{2} + {\mathbbm{a}}_{{12}} {\mathbb{A}\mathbb{B}} + {\mathbbm{a}}_{{13}} {\mathbb{B}\mathbb{C}} + {\mathbbm{a}}_{{23}} {\mathbb{A}\mathbb{C}}$$

42 $$Re_{x}^{{ - 1/2}} Nu_{x} = {\mathbbm{b}}_{0} + {\mathbbm{b}}_{1} {\mathbb{A}} + {\mathbbm{b}}_{2} {\mathbb{B}} + {\mathbbm{b}}_{3} {\mathbb{C}} + {\mathbbm{b}}_{{11}} {\mathbb{A}}^{2} + {\mathbbm{b}}_{{22}} {\mathbb{B}}^{2} +{\mathbbm{b}}_{{33}} {\mathbb{C}}^{2} + {\mathbbm{b}}_{{12}} {\mathbb{A}\mathbb{B}} + {\mathbbm{b}}_{{13}} {\mathbb{B}\mathbb{C}} + {\mathbbm{b}}_{{23}} {\mathbb{A}\mathbb{C}}$$

43 $$Re_{x}^{{ - 1/2}} Sh_{x} = {\mathbb{c}}_{0} + {\mathbb{c}}_{1} {\mathbb{A}} + {\mathbb{c}}_{2} {\mathbb{B}} +{\mathbbm{c}}_{3} {\mathbb{C}} + {\mathbbm{c}}_{{11}} {\mathbb{A}}^{2} + {\mathbbm{c}}_{{22}} {\mathbb{B}}^{2} + {\mathbbm{c}}_{{33}} {\mathbb{C}}^{2} + {\mathbbm{c}}_{{12}} {\mathbb{A}\mathbb{B}} + {\mathbbm{c}}_{{13}} {\mathbb{B}\mathbb{C}} + {\mathbbm{c}}_{{23}}{\mathbb{A}\mathbb{C}}$$

where, ${\mathbbm{a}}_{i}$, ${\mathbbm{b}}_{i}$, ${\mathbbm{c}}_{i}$, ${\mathbbm{a}}_{ij}$, ${\mathbbm{b}}_{ij}$ and ${\mathbbm{c}}_{ij}$ $i=j=1, 2, 3$ are an unidentified constants. The RSM can be used to find these unidentified coefficients. Nineteen degrees of freedom and twenty runs are needed for RSM, which are represented by three distinct parameters. Table 3 shows the levels of these parameters, symbols at low (− 1), medium (0), and highest (+ 1).The input parameters ($\phi$, ${M}_{r}$, $\delta$), ($\phi , {D}_{f}, Nr$), and ($\phi , {S}_{r}, {E}_{A}$), where $\phi ={\phi }_{1}+{\phi }_{2}+{\phi }_{3}$ for the wall stresses, Nusselt and Sherwood number for factors are and their responses are displayed in Table 4, Table 5 and Table 6, respectively.

**Table 3:** Design of experiment, the coded values, symbolic representation and diverse levels.

Levels				
Input parameter	Coded value	Low	Medium	High
$\left(0.1\le {M}_{r}\le 0.5\right)$	${\mathbb{A}}$	$0.1$	$0.25$	$0.5$
$\left(1\%\le \phi \le 5\%\right)$	${\mathbb{B}}$	$1\%$	$2.5\%$	$5\%$
$\left(0.1\le \delta \le 0.5\right)$	${\mathbb{C}}$	$0.1$	$0.25$	$0.5$
Levels				
Input parameter	Coded value	Low	Medium	High
$\left(0.1\le {D}_{f}\le 0.5\right)$	${\mathbb{A}}$	$0.1$	$0.25$	$0.5$
$\left(1\%\le \phi \le 5\%\right)$	${\mathbb{B}}$	$1\%$	$2.5\%$	$5\%$
$\left(0.1\le Nr\le 0.5\right)$	${\mathbb{C}}$	$0.1$	$0.25$	$0.5$
Levels				
Input parameter	Coded value	Low	Medium	High
$\left(0.1\le {S}_{r}\le 0.5\right)$	${\mathbb{A}}$	$0.1$	$0.25$	$0.5$
$\left(1\%\le \phi \le 5\%\right)$	${\mathbb{B}}$	$1\%$	$2.5\%$	$5\%$
$\left(0.1\le {E}_{A}\le 0.5\right)$	${\mathbb{C}}$	$0.1$	$0.25$	$0.5$

**Table 4:** Experimental scheme with coded, actual values and response of ${Re}_{x}^{1/2}{C}_{f}$.

Runs	Coded variables			Real parameters			Response
	${\mathbb{A}}$	${\mathbb{B}}$	${\mathbb{C}}$	${M}_{r}$	$\phi$	$\delta$	${Re}_{x}^{1/2}{C}_{f}$
1	$-1$	$-1$	$-1$	0.1	1%	0.1	$-$ 2.03455
2	$-1$	$-1$	1	0.1	1%	0.05	$-$ 1.97560
3	$-1$	1	$-1$	0.1	2.5%	0.1	$-$ 1.00698
3	1	$-1$	$-1$	0.5	5%	0.5	$-$ 2.05356
4	$-1$	1	1	0.1	5%	0.5	$-$ 2.54774
5	1	$-1$	1	0.5	1%	0.5	$-$ 2.95633
6	1	1	$-1$	0.5	5%	0.1	$-$ 2.03456
7	1	1	1	0.5	5%	0.5	$-$ 2.13409
8	0	$-1$	$-1$	0.25	1%	0.1	$-$ 2.24940
9	$-1$	0	$-1$	0.1	2.5%	0.1	$-$ 2.03456
10	$-1$	$-1$	0	0.1	1%	0.25	$-$ 1.89643
11	0	$-1$	$-1$	0.25	1%	0.1	$-$ 1.24548
12	$-1$	0	0	0.1	2.5%	0.25	$-$ 2.03464
13	0	$-1$	0	0.25	1%	0.25	$-$ 1.05632
14	1	0	0	0.5	2.5%	0.25	$-$ 1.00541
15–20	0	1	0	0.25	5%	0.25	$-$ 2.03675

**Table 5:** Experimental scheme with coded, actual values and response ${Re}_{x}^{-1/2}{Nu}_{x}$.

Runs	Coded variables			Real parameters			Response
	${\mathbb{A}}$	${\mathbb{B}}$	${\mathbb{C}}$	$Nr$	$\phi$	${D}_{f}$	${Re}_{x}^{-1/2}{Nu}_{x}$
1	$-1$	$-1$	$-1$	0.1	1%	0.1	0.2280
2	$-1$	$-1$	1	0.1	1%	0.05	0.2466
3	$-1$	1	$-1$	0.1	2.5%	0.1	0.2619
3	1	$-1$	$-1$	0.5	5%	0.5	0.2590
4	$-1$	1	1	0.1	5%	0.5	0.2684
5	1	$-1$	1	0.5	1%	0.5	0.2901
6	1	1	$-1$	0.5	5%	0.1	0.2871
7	1	1	1	0.5	5%	0.5	0.3278
8	0	$-1$	$-1$	0.25	1%	0.1	0.3576
9	$-1$	0	$-1$	0.1	2.5%	0.1	0.1096
10	$-1$	$-1$	0	0.1	1%	0.25	0.0964
11	0	$-1$	$-1$	0.25	1%	0.1	0.0845
12	$-1$	0	0	0.1	2.5%	0.25	0.2708
13	0	$-1$	0	0.25	1%	0.25	0.3265
14	1	0	0	0.5	2.5%	0.25	0.3460
15–20	0	1	0	0.25	5%	0.25	0.2280

**Table 6:** Experimental scheme with coded values, actual values and response of ${Re}_{x}^{-1/2}{Sh}_{x}.$.

Runs	Coded variables			Real parameters			Response
	${\mathbb{A}}$	${\mathbb{B}}$	${\mathbb{C}}$	${S}_{r}$	$\phi$	${E}_{A}$	${Re}_{x}^{-1/2}{Sh}_{x}$
1	$-1$	$-1$	$-1$	0.1	1%	0.1	0.1534
2	$-1$	$-1$	1	0.1	1%	0.05	0.1692
3	$-1$	1	$-1$	0.1	2.5%	0.1	0.1803
3	1	$-1$	$-1$	0.5	5%	0.5	0.1793
4	$-1$	1	1	0.1	5%	0.5	0.1944
5	1	$-1$	1	0.5	1%	0.5	0.2050
6	1	1	$-1$	0.5	5%	0.1	0.1730
7	1	1	1	0.5	5%	0.5	0.1890
8	0	$-1$	$-1$	0.25	1%	0.1	0.1931
9	$-1$	0	$-1$	0.1	2.5%	0.1	0.1435
10	$-1$	$-1$	0	0.1	1%	0.25	0.1069
11	0	$-1$	$-1$	0.25	1%	0.1	0.0915
12	$-1$	0	0	0.1	2.5%	0.25	0.2087
13	0	$-1$	0	0.25	1%	0.25	0.2241
14	1	0	0	0.5	2.5%	0.25	0.2315
15–20	0	1	0	0.25	5%	0.25	0.1534

The revised fitted equations are re-written as:

44 $$\begin{aligned} \mathrm{Re} _{x}^{{1/2}} C_{f} & = 3.0542 + 0.8816M_{r} + 0.1126\phi - 0.1056\delta + 0.00086M_{r}^{2} \\ & + 0.0743\phi ^{2} + 0.0479\delta ^{2} - 0.00175M_{r} \phi - 0.02615\phi \delta - 0.0731\delta M_{r} \\ \end{aligned}$$

45 $$\begin{aligned} \mathrm{Re} _{x}^{{ - 1/2}} Nu_{x} & = 0.7856 + 0.1441D_{f} - 2.780\phi - 0.2908Nr + 0.00532D_{f}^{2} + 3.645\phi ^{2} \\ & + 0.0678Nr^{2} - 0.2273D_{f} \phi - 0.03699\phi Nr + 0.6147NrD_{f} \\ \end{aligned}$$

46 $$\begin{aligned} \mathrm{Re} _{x}^{{ - 1/2}} Sh_{x} & = - 2.09176 + 2.0786S_{r} - 3.08678\phi + 1.2873E_{A} + 2.07862S_{r}^{2} \\ & + 0.09073\phi ^{2} + 0.26367E_{A}^{2} - 1.89654S_{r} \phi - 0.16474\phi E_{A} + 0.2365S_{r} E_{A} \\ \end{aligned}$$

### Analysis of variance (ANOVA) test

The statistical technique known as ANOVA leverages variance to identify statistically significant interactions among multiple input variables. Table 4, Table 5 and Table 6, displays the regression model results for twenty runs, which are calculated using the statistical program MINITAB-19. To get these results, the sequential F-test and ANOVA was used. ANOVA-generated statistical estimators are all displayed in Tables 5 and 6. The F-value and p-value for Eq. (41), (42) and (43) are distinct from one another. To determine the variance of the data, the F-value is utilized. The accuracy of the input data is acknowledge if the F-value is greater than one. Regression model validation is another application which is obtained from the p-value. A statistically significant p-value is less than 0.05, and a p-value above 0.05 is invalid and will not be taken into account when constructing the output response equation.

Table 7, display the ANOVA results for wall stresses, namely the skin friction ${Re}_{x}^{1/2}{C}_{f}$, Sherwood number ${Re}_{x}^{-1/2}{Sh}_{x}$ for mass transfer rate, and Nusselt ${Re}_{x}^{-1/2}{Nu}_{x}$ for heat transfer rate. These tables present a range of test findings, comprising diverse sources and their degrees of freedom, and related metrics such as the $p-$ values and $F-$ value and total error. A statistical threshold of 5%, or a 95% confidence level, is often reached when the $F-$ value is greater than 1 and the $p-$ values are less than 0.05. The accuracy of the model is improved by fulfilling these requirements. Fascinatingly, the ANOVA-derived p-values span the whole range of the three parameters taken in the analysis. Furthermore, a model that incorporates the shear rate and Nusselt and sherwood numbers are suggested in order to represent the conclusion. Both the computed and adjusted values in the resulting table produce ${R}^{2}=98.86\%$, Adj ${R}^{2}=98.90\%$ for ${Re}_{x}^{1/2}{C}_{f}$, ${R}^{2}=99.99\%$, Adj ${R}^{2}=99.95\%$ for ${Re}_{x}^{-1/2}{Nu}_{x}$ and ${R}^{2}=99.87\%$, Adj ${R}^{2}=99.91\%$ for ${Re}_{x}^{-1/2}{Sh}_{x}$, respectively. According to this a nutshell the suggested model most closely matches the responses provided. When a model term p-value is less than 0.0500, it is generally regarded as significant; when it is greater than 0.1000, it is deemed insignificant. Therefore, model reduction could help a model perform better if it has a lot of extraneous terms.

**Table 7:** ANOVA test ustilzing qurdatic models for ${Re}_{x}^{1/2}{C}_{f}$, ${Re}_{x}^{-1/2}{Nu}_{x}$ and ${Re}_{x}^{-1/2}{Sh}_{x}$.

$${Re}_{x}^{1/2}{C}_{f}$$						
Source	DF	SS	MS	F-test	p-value	
Model	9	544.09	14.840	525.10	$<$ 0.0000	Significant
${M}_{r}$	1	54.56	8.56	2.280	$<$ 0.0000	
$\phi$	1	567.45	5.42	73.079	$<$ 0.0000	
$\delta$	1	73.6	2.98	108.089	$<$ 0.0001	
${M}_{r}\times \phi$	1	406.2	16.16	80.478	$<$ 0.0000	
$\delta \times \phi$	1	20.78	0.654	0.281	0.65437	Insignificant
$\delta \times {M}_{r}$	1	9.78	1.03	0.2205	0.45960	Insignificant
${M}_{r}^{2}$	1	70.7	2.470	109.13	$<$ 0.0000	Significant
${\phi }^{2}$	1	7.75	0.0856	131.09	$<$ 0.0000	
${\delta }^{2}$	1	15.20	0.0674	0.904	0.69037	Insignificant
Residual	10	0.2267	1.565	$-$	$-$	
Lack of Fit	5	0.00085	0.0005	$-$	$-$	
Actual Error	5	0.00000	0.00000			
Total	19	35.1340	$-$	$-$	$-$	
$S=0.0903$, ${R}^{2}=98.86\%$, Adj ${R}^{2}=98.90\%$						
$${Re}_{x}^{-1/2}{Nu}_{x}$$						
Source	DF	SS	MS	F-test	p-value	
Model	9	1593.49	198.87	789.90	$<$ 0.0001	Significant
${D}_{f}$	1	402.62	7.697	3.098	$<$ 0.0001	
$\phi$	1	59.56	4.29	90.0943	$<$ 0.0000	
$Nr$	1	456.0	8.81	134.31	$<$ 0.0000	
${D}_{f}\times \phi$	1	782.08	34.012	0.789	0.34532	Insignificant
$Nr\times \phi$	1	34.86	2.5409	1.456	$<$ 0.0001	Significant
$Nr\times {D}_{f}$	1	45.80	10.25	45.13	$<$ 0.0001	Significant
${D}_{f}^{2}$	1	23.89	3.092	0.48	0.67892	Insignificant
${\phi }^{2}$	1	45.32	0.8612	30.87	$<$ 0.0000	Significant
${Nr}^{2}$	1	29.340	1.1645	1.0456	$<$ 0.0000	Significant
Residual	10	0.2267	1.565	$-$	$-$	
Lack of Fit	5	0.00085	0.0005	$-$	$-$	
Pure Error	5	0.00000	0.00000			
Total	19	19.8934	$-$	$-$	$-$	
$S=0.0993$, ${R}^{2}=99.99\%$, Adj ${R}^{2}=99.95\%$						
$${Re}_{x}^{-1/2}{Sh}_{x}$$						
Source	DF	SS	MS	F-test	p-value	
Model	9	467.56	2.8967	345.56	$<$ 0.0000	Significant
${S}_{r}$	1	108.78	9.690	5.8090	$<$ 0.0001	
$\phi$	1	69.32	8.236	23.093	$<$ 0.0000	
${E}_{A}$	1	34.5	3.06	56.876	$<$ 0.0001	
${S}_{r}\times \phi$	1	489.1	6.873	0.789	0.65437	Insignificant
${E}_{A}\times \phi$	1	3.672	0.9980	1.21982	$<$ 0.0000	Significant
${E}_{A}\times {S}_{r}$	1	12.8983	2.454	1.2356	$<$ 0.0000	
${S}_{r}^{2}$	1	21.4	4.3443	92.356	$<$ 0.0000	
${\phi }^{2}$	1	2.556	1.8056	0.4895	0.5563	Insignificant
${E}_{A}^{2}$	1	24.20	2.4560	1.096	$<$ 0.0001	
Residual	10	0.3798	2.6053	$-$	$-$	
Lack of Fit	5	0.00055	0.00023	$-$	$-$	
Pure Error	5	0.00000	0.00000			
Total	19	12.8565	$-$	$-$	$-$	
$S=0.09823$, ${R}^{2}=99.87\%$, Adj ${R}^{2}=99.91\%$						

**Fig. 15:**
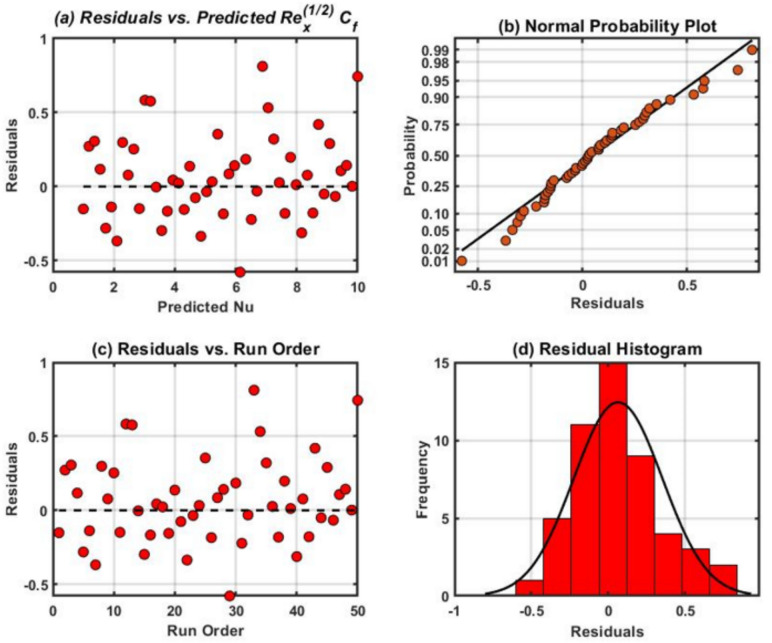
Residual plots for ${Re}_{x}^{1/2}{C}_{f}.$.

**Fig. 16:**
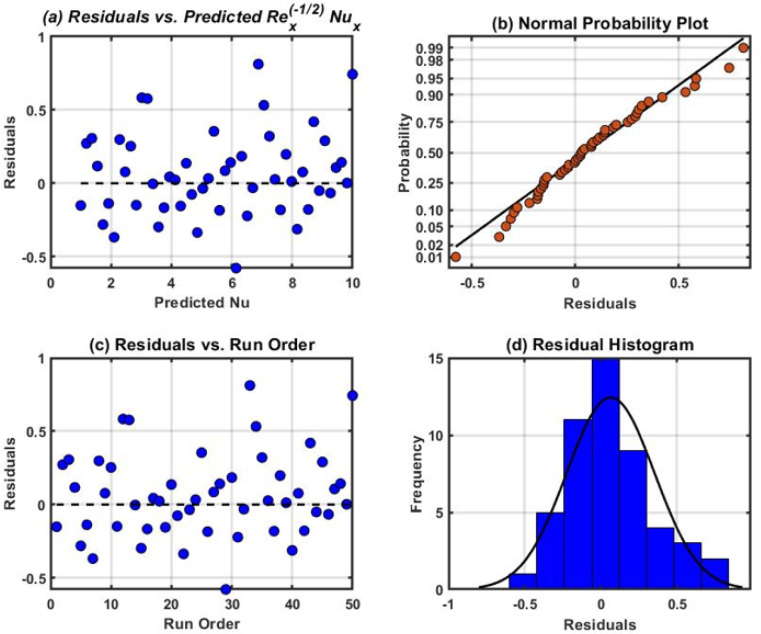
Residual plots for ${Re}_{x}^{-1/2}{Nu}_{x}.$.

The residual plots demonstrated in Figs. 14, 15, and 16 were attained for $${Re}_{x}^{1/2}{C}_{f}$$, $${Re}_{x}^{-1/2}{Nu}_{x}$$ and $${Re}_{x}^{-1/2}{Sh}_{x}$$. These graphs demonstrate the good state of normal probability plots. The normal probability plot, is a visualization tool used to evaluate a dataset normality or compare it to a speculative normal distribution. One can ascertain whether or not the data follow an average (Gaussian) distribution by looking at the plot visually. A comparable theoretical quantile from the conventional normal distribution is assigned to each data point in the dataset. If the data were perfectly regularly distributed, the plot straight diagonal line would reveal what would happen. Furthermore, residual histograms resemble symmetrical distributions more and have less skew. A strong correlation among observed and fitted values was demonstrated by comparing the residual diagrams and fitted values. The highest residuals for the mean $$Re_{x}^{1/2} C_{f}$$$$,$$ and $$Re_{x}^{ - 1/2} Sh_{x}$$, respectively is found to be in the adjacency of 0.25 and 0.50 in all cases.

### Sensitivity analysis (SA)

SA is a robust statistical approach which is used to evaluate which distinct inputs affect a model output^56^. A common method for measuring sensitivity to model variables is to derive the response function. Important proof of response effectiveness is provided by SA which led to the development of contemporary control theory, which incorporates synthesis, optimization, and adaptation. The concept of SA is used in chemical engineering, meta-analyses, atmospheric geophysics, nuclear engineering, biological sciences, and engineering domains. In this study, the sensitivity of the skin friction, Nusselt and Sherwood numbers, to the input parameters ($\phi$, ${M}_{r}$, $\delta$), ($\phi , {D}_{f}, Nr$), and ($\phi , {S}_{r}, {E}_{A}$), where $\phi ={\phi }_{1}+{\phi }_{2}+{\phi }_{3}$ were examined. The sensitivity is computed utilizing regression Eq. (45), (46) and (47). The sensitivity functions are estimated using partial derivatives of the response output with respect to the input variables give below.

47 $$\frac{{\partial \left( {Re_{x}^{1/2} C_{f} } \right)}}{{\partial M_{r} }} = 0.8816 + 0.000172M_{r} - 0.00175\phi - 0.0731\delta$$

48 $$\frac{{\partial \left( {Re_{x}^{1/2} C_{f} } \right)}}{\partial \phi } = 0.1126 + 0.1486\phi - 0.00175M_{r} - 0.02615\delta$$

49 $$\frac{{\partial \left( {Re_{x}^{1/2} C_{f} } \right)}}{\partial \delta } = - 0.1056 + 0.0958\delta - 0.02615\phi - 0.0731M_{r}$$

50 $$\frac{{\partial \left( {Re_{x}^{ - 1/2} Nu_{x} } \right)}}{{\partial D_{f} }} = 0.1441D_{f} + 0.01064D_{f} - 0.2273\phi + 0.6147D_{f}$$

51 $$\frac{{\partial \left( {Re_{x}^{ - 1/2} Nu_{x} } \right)}}{\partial \phi } = - 2.780 + 7.29\phi - 0.2273D_{f} - 0.03699Nr$$

52 $$\frac{{\partial \left( {Re_{x}^{ - 1/2} Nu_{x} } \right)}}{\partial Nr} = - 0.2908 + 0.1356Nr - 0.03699\phi + 0.6147D_{f}$$

53 $$\frac{{\partial \left( {Re_{x}^{ - 1/2} Sh_{x} } \right)}}{{\partial S_{r} }} = 2.0786 + 4.15724S_{r} - 1.89654\phi + 0.2365E_{A}$$

54 $$\frac{{\partial \left( {Re_{x}^{ - 1/2} Sh_{x} } \right)}}{\partial \phi } = - 3.08678 + 0.18146\phi - 1.89654S_{r} - 0.16474E_{A}$$

55 $$\frac{{\partial \left( {Re_{x}^{ - 1/2} Sh_{x} } \right)}}{{\partial E_{A} }} = 1.2873 + 0.52734E_{A} - 0.16474\phi + 0.2365S_{r}$$

The sensitivity of $$Re_{x}^{1/2} C_{f}$$, $$Re_{x}^{ - 1/2}$$$$Nu_{x}$$, and $$Re_{x}^{ - 1/2} Sh_{x}$$ are displayed in Table 8, Table 9 and Table 10, respectively. If the component bar chart is positive, the response increases; if it is negative, the response decreases. As the peak of the bar chart increases, the responses become more responsive to the input elements. Figure 3 and 4 display the sensitivity graphs for $${Re}_{x}^{1/2}{C}_{f}$$, $${Re}_{x}^{-1/2}{Nu}_{x}$$, and $$Re_{x}^{ - 1/2} Sh_{x}$$, respectively.

**Table 8:** Sensitivity of ${Re}_{x}^{1/2}{C}_{f}$ with response variables setting ${M}_{r}=0$.

$\phi$	$\delta$	$\frac{\partial \left({Re}_{x}^{1/2}{C}_{f}\right)}{\partial \phi }$	$\frac{\partial \left({Re}_{x}^{1/2}{C}_{f}\right)}{\partial \phi }$	$\frac{\partial \left({Re}_{x}^{1/2}{C}_{f}\right)}{\partial \delta }$
$-1$	$-1$	2.3165	1.6284	1.2092
	0	2.4533	2.8745	2.3456
	1	1.9808	$-$ 2.8286	2.8445
0	$-1$	2.8307	2.7784	2.8156
	0	1.5726	$-$ 2.1905	1.9893
	1	2.0202	2.4614	1.8879
1	$-1$	1.9826	2.6903	$-$ 2.2898
	0	1.3728	$-$ 1.9989	$-$ 2.6270
	1	1.5704	1.8092	$-$ 1.8907

**Table 9:** Sensitivity of ${Re}_{x}^{-1/2}{Nu}_{x}$ with response variables setting ${D}_{f}=0$.

$\phi$	$Nr$	$\frac{\partial \left({Re}_{x}^{-1/2}{Nu}_{x}\right)}{\partial {D}_{f}}$	$\frac{\partial \left({Re}_{x}^{-1/2}{Nu}_{x}\right)}{\partial \phi }$	$\frac{\partial \left({Re}_{x}^{-1/2}{Nu}_{x}\right)}{\partial Nr}$
$-1$	$-1$	$-$ 0.6545	0.8487	0.2289
	0	$-$ 0.5830	0.9534	0.8789
	1	$-$ 0.4535	0.6443	0.4482
0	$-1$	0.6784	0.7648	0.5623
	0	0.9830	0.9345	0.9345
	1	0.6982	0.6904	0.8794
1	$-1$	0.7954	0.8943	$-$ 0.8434
	0	0.8430	0.8909	$-$ 0.2704
	1	0.5463	0.8429	$-$ 0.9070

**Table 10:** Sensitivity of ${Re}_{x}^{-1/2}{Sh}_{x}$ with response variables setting ${S}_{r}=0$.

$\phi$	${E}_{A}$	$\frac{\partial \left({Re}_{x}^{-1/2}{Sh}_{x}\right)}{\partial {S}_{r}}$	$\frac{\partial \left({Re}_{x}^{-1/2}{Sh}_{x}\right)}{\partial \phi }$	$\frac{\partial \left({Re}_{x}^{-1/2}{Sh}_{x}\right)}{\partial {E}_{A}}$
$-1$	$-1$	0.1023	$-$ 0.1470	0.1234
	0	0.1835	$-$ 0.1580	0.1365
	1	0.1233	$-$ 0.1438	0.1420
0	$-1$	0.1744	0.1688	0.1639
	0	0.1306	0.1357	0.1359
	1	0.1825	0.1656	0.1740
1	$-1$	0.1744	0.1890	0.1424
	0	0.1445	0.1096	0.1742
	1	0.1439	0.1495	0.1706

**Fig. 17:**
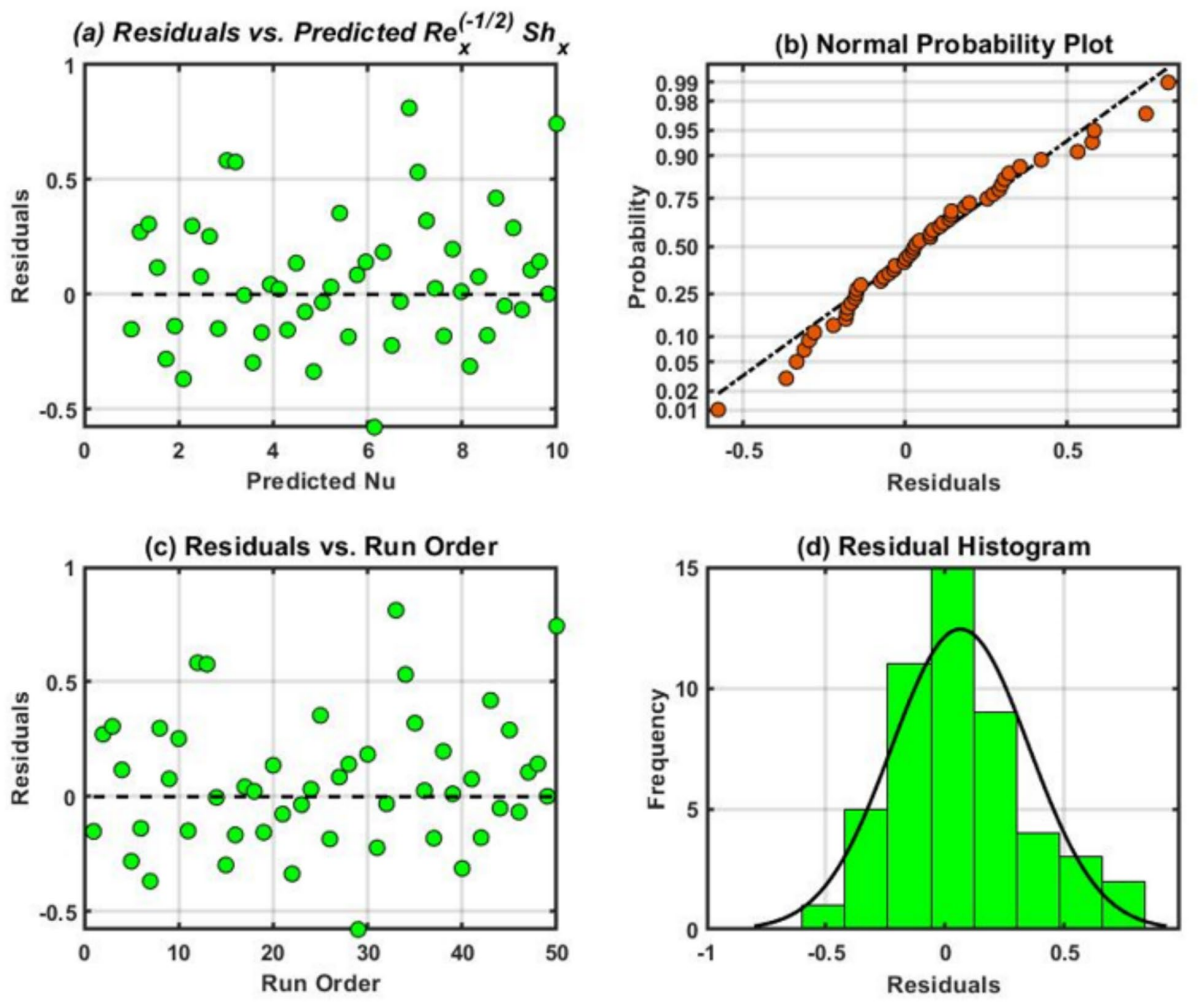
Residual plots for ${Re}_{x}^{-1/2}{Sh}_{x}.$.

**Fig. 18:**
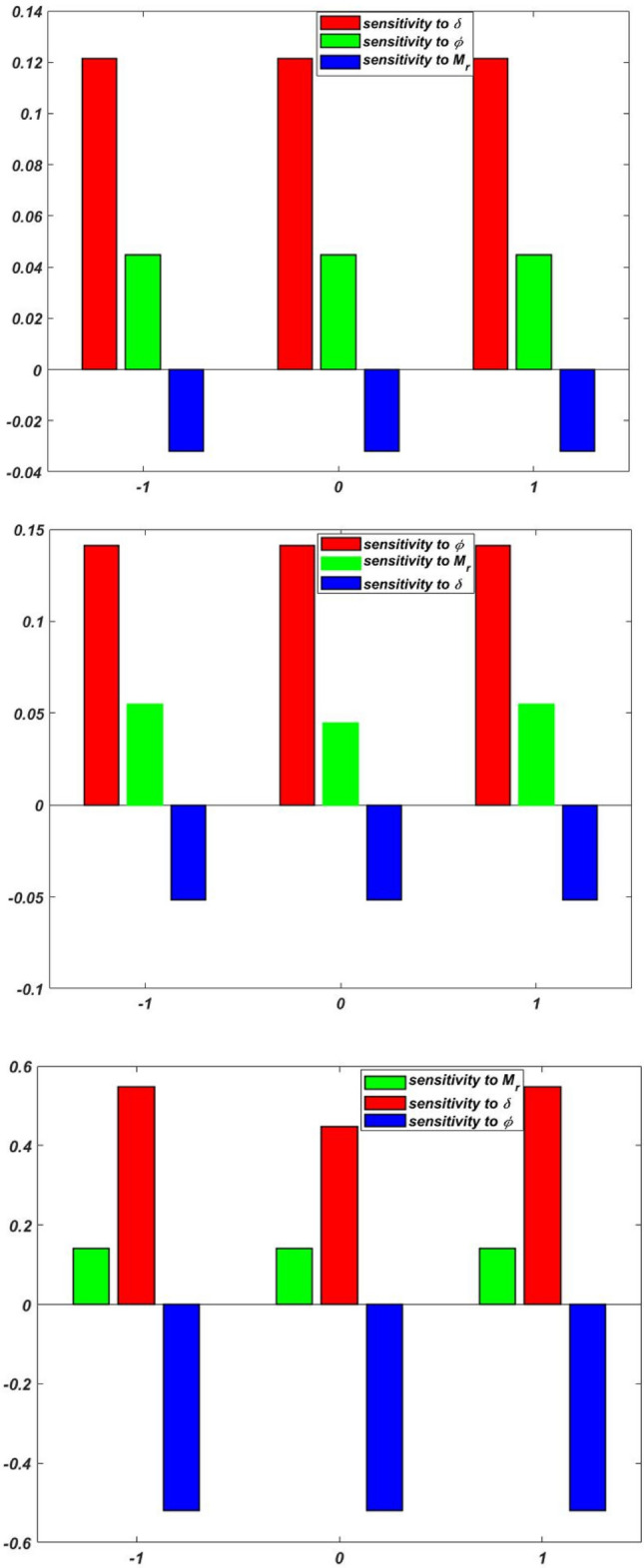
Sensitivity layout for ${Re}_{x}^{1/2}{C}_{f}$ in a scenario (a) $\phi =-1$ (b) $\phi =0$ (c) $\phi =1$.

**Fig. 19:**
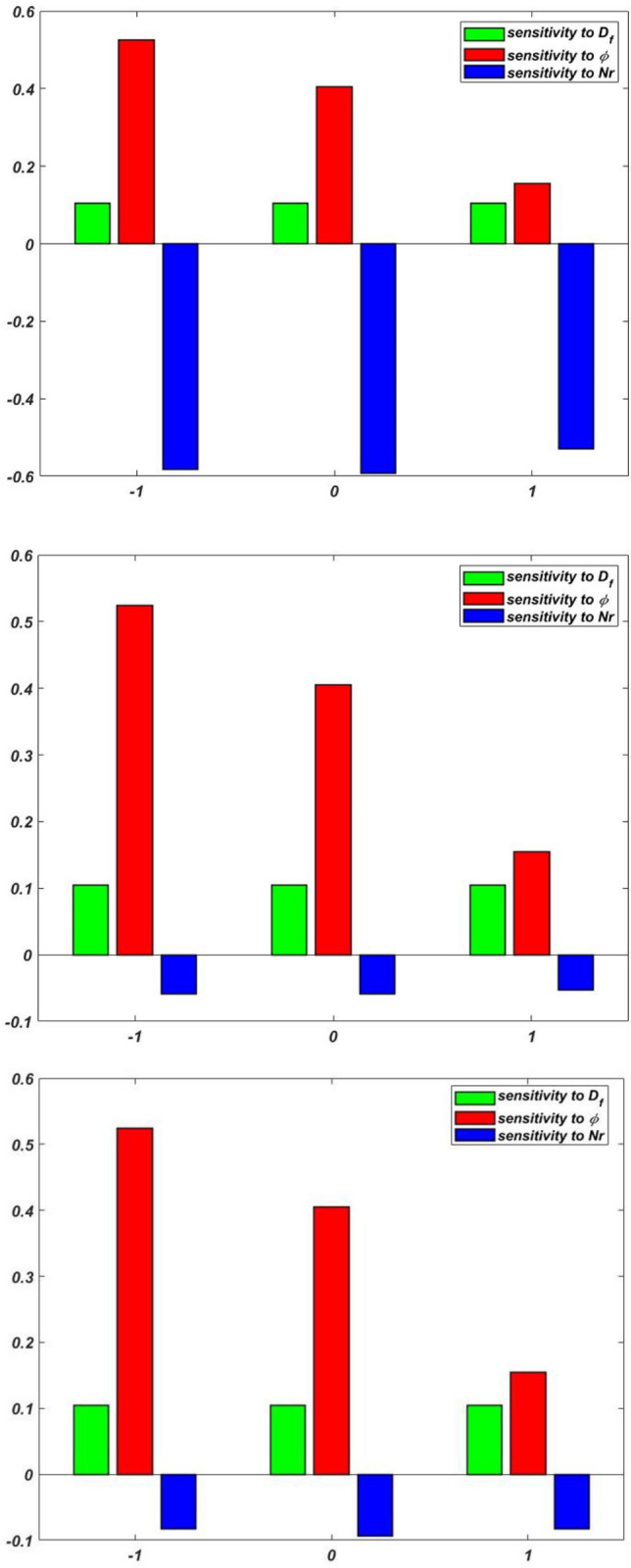
Sensitivity layout for ${Re}_{x}^{-1/2}{Nu}_{x}$ in a scenario (a) $\phi =-1$ (b) $\phi =0$ (c) $\phi =1$.

For more significant insight, the results of the SA have been displayed via vertical bar charts in Fig. 17, 18, 19, and 20. The concentration of tri-nanoparticles $\phi$ have a significant impact on the sensitivity of $Re_{x}^{1/2} C_{f}$. The leading positive response $\frac{{\partial \left( {Re_{x}^{1/2} C_{f} } \right)}}{\partial \phi }$ of 2.8307 at $M_{r} = 0$ is found at $\phi =0$ and $\delta =0$, suggesting that the insertion of three nanoparticles in thin BL increases friction (Fig. 18). At $\phi =1$ and $\delta =1$, the negative sensitivitiy of $-$ 2.8286 appear, indicating that the buildup of nanoparticles can lessen the friction in thick layers. At low nanoparticle concentrations, the BL thickness dominates the friction effects, as seen by positve values $\delta$. The sensitivity 2.8445 peaking at $\phi =-1$ and $\delta =1$.

**Fig. 20:**
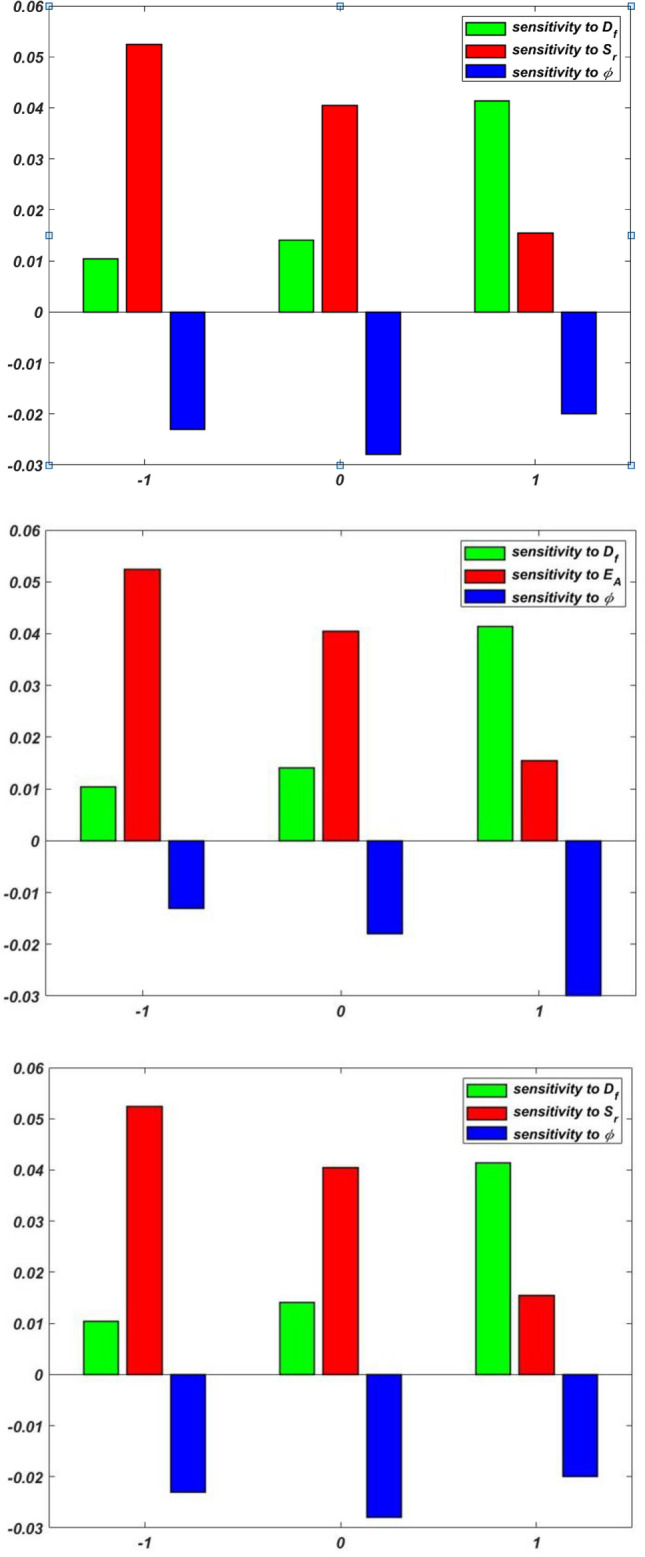
Sensitivity layout for ${Re}_{x}^{-1/2}{Sh}_{x}$ in a scenario (a) $\phi =-1$ (b) $\phi =0$ (c) $\phi =1$.

The SA of ${Re}_{x}^{-1/2}{Nu}_{x}$ taking ${D}_{f}=0$, identifies three important trends in the patterns of heat transport as shown in Fig. 19. The first is that the concentration of nanoparticles $\phi$ which improves the thermal performance. The findings shows positive sensitivity of $\frac{{\partial \left( {Re_{x}^{ - 1/2} Nu_{x} } \right)}}{\partial \phi }$ in all situations, peaking at 0.9534 for $\phi =-1$ and $Nr=0$. This suggests that the highest thermal improvement happens at low nanoparticle load with moderate thermal radiation. Secondly, there is a shift from positive to negative influence in the thermal radiation parameter $Nr$. While low $\phi$ conditions $-1\le \phi \le 0$ suggest an increased heat transfer $\frac{\partial \left({Re}_{x}^{-1/2}{Nu}_{x}\right)}{\partial Nr}$ with radiation up to 0.9345 at $\phi =0$, $Nr=1$. On the other hand, high nanoparticle concentrations $\phi =1$ exhibit reverse trends with a substantial negative sensitivity $-$ 0.9070 at $\phi =1$, $Nr=1$, indicating that nanoparticle clustering may obstruct radiative heat transfer. Thirdly, the cross-derivative $\frac{\partial \left({Re}_{x}^{-1/2}{Nu}_{x}\right)}{\partial {D}_{f}}$ changes from unfavorable to positive results as $\phi$ increases, reaching its highest positive sensitivity of 0.8430 at $\phi =1$, $Nr=0$. This indicates that the sum of the effects of thermal radiation and nanoparticle concentration produces the optimal conditions for improving heat transfer at adequate parameter values.

The SA of ${Re}_{x}^{-1/2}{Sh}_{x}$ is displayed in Fig. 20 for three distinct scenarios. Mass transfer at low concetration $\phi =-1$, exhibits a negative sensitivity to nanoparticle concentration $\frac{\partial \left({Re}_{x}^{-1/2}{Sh}_{x}\right)}{\partial \phi }$ ranging from $-$ 0.1580 to $-$ 0.1438 but a positive response is achived against activation energy $\frac{\partial \left({Re}_{x}^{-1/2}{Sh}_{x}\right)}{\partial {E}_{A}}$ ranges between 0.1234 to 0.1420, indicating that the addition of nanoparticles reduces the mass transfer while adding more activation energy increases it. The highest sensitivity of 0.1688 at $\phi =-1$, and $\frac{\partial \left({Re}_{x}^{-1/2}{Sh}_{x}\right)}{\partial {E}_{A}}$ of 0.1639 occur at ${E}_{A}=-1$, while all sensitivities turn positive when $\phi =0$, moderate concentration. The high concentration regime $\phi =1$) exhibits peak sensitivity $\frac{\partial \left({Re}_{x}^{-1/2}{Sh}_{x}\right)}{\partial {E}_{A}}$ to both activation energy of 0.1742 at ${E}_{A}=0$ and nanoparticle concentration $\frac{\partial \left({Re}_{x}^{-1/2}{Sh}_{x}\right)}{\partial \phi }=$ 0.1890 at ${E}_{A}=-1,$ suggesting that optimal mass transfer enhancement happens when high nanoparticle loading and moderate activation energy are combined. The derivative of Soret number $\frac{\partial \left({Re}_{x}^{-1/2}{Sh}_{x}\right)}{\partial {S}_{r}}$ is always positive under all situations ranges from 0.1023 to 0.1835, showing that the Soret number has a positive impact on mass transfer, with its greatest effect at $\phi =-1$ and ${E}_{A}=0$.

## Conclusions

The study scrutinized the unsteady BLF with energy and mass transport of viscous water-based tri-HNF over a Riga plate. The Riga sensor consist of permanent magnets and electrodes, while the tri-HNF is made up of $A{l}_{2}{O}_{3}$, $CuO$ and $Ti{O}_{2}$ nanoparticles. A statistical technique RSM, was used to optimize the wall drag, energy and mass transfer rate. Variations in the various parameters were found to significantly alter the fluid flow patterns as well as the heat and transfer performance. In this model, the RSM worked well for evaluating and improving the shear and heat transfer rates. SA of various independent parameters on output responses is carried out using these numerical and statistical values. The relationship between input and output responses/variables has been established with deployment of RSM. Using ANOVA, the residual errors and regression coefficient are also computed. To investigate the SA of various independent input parameters on dependent output responses, these numerical and statistical results are utilized. The study significant findings are listed as:

- Flow profile near stagnation region uplift with ratio parameter $A$, while opposite behavior was observed for volumetric load of nanomaterials ${\phi }_{1}$, ${\phi }_{2}$ and ${\phi }_{3}$. The upward slope shows that the fluid velocity overcomes the free stream velocity at a specific point inside the BL.
- Velocity of the tri-HNF boost with modified Hartman number ${M}_{r}$ and space between magnets and electrodes parameter $\delta$.
- The temperature of tri-HNF augmented with radiation $Nr$, Dufour ${D}_{f}$, nanomaterial load and Biot number ${Bi}_{2}$. The upward slope shows that the fluid velocity overcomes the free stream velocity at a specific point inside the BL.
- The concentration agument as with an uptick in nanoparticle load, activation energy ${E}_{A}$, and $Sr$. The concentration profile increased in response to an increase in the activation energy and Biot number ${Bi}_{2}$, suggesting that the solute species were more widely distributed in the fluid. In contrast, an increase in $Sc$ led to a decreasing concentration profile, indicating the influence of chemical kinetics and diffusion on the fluid solute distribution.
- The tri-HNF entire heat transfer efficiency was further improved by the synergistic impacts of the selected nanoparticle combination.
- The SA illustrates that tri-hybrid nanoparticles substantially raises the skin friction at $\phi =0$, $\delta =0$, in thin BL, while reducing the friction for $\phi =0$, $\delta =1$ in thick BL. This indicates that the thickness of the BL plays a crucial role in controlling the effects of nanoparticles on wall shear stress.
- Under low nanoparticle loading with moderate radiation, the thermal transport sensitivity shows maximum enhancement at $\phi =-1$, $Nr=0.$ However, at $\phi =1$, $Nr=1$, high concentrations together with strong radiation cause heat transfer to deteriorate, indicating an ideal balance between thermal radiation and nanoparticle concentration for peak thermal performance.
- Nanoparticle loading thresholds control the transition among mass transfer reduction and improvement. At low nanoparticle loading, the mass transfer exhibits concentration-dependent behavior, exhibiting negative sensitivity that reverses to a robust positive response at high concentrations. At $\phi =1$, ${E}_{A}$, an activation energy frequently boosts mass transfer.
- Statistical technique using RSM combines with ANOVA shows optimized rate of coefficients such as shear rate and heat and mass transfer rate for the proposed factors.
- In BLF, an activation energy and cross-diffusion mechanisms provide crucial mass transfer complications. A higher EA indicates that chemical reactions need more thermal energy to begin, which reduces reaction rates near the BL cooler outer edges and concentrates mass transfer closer to the heated surface, decreasing the concentration gradient.
- The Soret effect causes nanoparticles to migrate from hot to cold areas because of a temperature gradient, whereas the Dufour effect produces a heat flux because of a concentration gradient. These effects are crucial in practical electromagnetic flow control systems, such as those employing Riga plates.
- The Soret effect can be used in sophisticated cooling systems for nuclear or spacecraft parts to prevent nanoparticle deposition on hot surfaces, and controlling activation energy is essential for forecasting chemical accumulation or corrosion rates in electrochemical processes regulated by electromagnetic fields, which ultimately affects the system’s thermal performance and material longevity.

Future research could examine the dynamics of other nanoparticle and base fluid combinations to find even more energy-efficient tri-HNF compositions. The model can be expanded to include more intricate geometries, such as curved or wavy Riga surfaces, and three-dimensional flow arrangements. Additionally, adding sophisticated machine learning algorithms to RSM could improve the mass and heat transfer rates’ optimization efficiency and predictive accuracy, offering a solid foundation for the clever design of next-generation thermal control systems.

## Data Availability

The author confirms that the data supporting the findings of this study are available within the manuscript.
